# Process evaluation of a complex intervention to improve the oral health of nursing home residents (MundZaRR): a multimethod study

**DOI:** 10.1186/s12877-026-08607-z

**Published:** 2026-11-05

**Authors:** Lisa-Maria Wobst, Gabriele Meyer, Sarah Habig, Saskia Gabelmann, Katrin Hertrampf, Georg Gaßmann, Falk Schwendicke, Peter Schlattmann, Helmut Orawa, Jens Abraham

**Affiliations:** 1 Institute of Health, Midwifery and Nursing Science, Medical Faculty of Martin Luther University Halle-Wittenberg, University Medicine Halle, Halle (Saale), Germany; 2 Clinic of Oral and Maxillofacial Surgery, University Hospital Schleswig-Holstein, Kiel, Germany; 3 praxisHochschule, University of Applied Sciences, Cologne, Germany; 4 Clinic for Conservative Dentistry and Periodontology, LMU University Hospital, LMU Munich, Munich, Germany; 5 Institute for Medical Statistics, Computer and Data Sciences, University Hospital Jena, Jena, Germany

**Keywords:** Process evaluation, Complex intervention, Nursing home, Oral health, Nursing home residents, Dental assistants

## Abstract

**Background:**

The oral health of nursing home residents is often poor. In the cluster-randomised controlled MundZaRR trial, a complex intervention to improve oral health was investigated across 18 nursing homes (*n* = 271 participants). A comprehensive process evaluation aimed to explore the experiences of various target groups and gain insights into the implementation of the intervention.

**Methods:**

The process evaluation followed international guidelines. The study was conducted in Germany and the data collection took place between July 2022 and October 2023 and comprised 23 individual, semi-structured interviews and three focus groups with dentists, dental assistants, nursing home managers and staff, and members of the resident council, 20 participatory observations, written surveys, structured protocols and document analysis. Quantitative data were analysed descriptively; qualitative data were analysed using content analysis.

**Results:**

Three educational training courses over approximately nine hours for dentists and dental assistants were performed largely as planned and evaluated positively. The intervention could not be implemented in one out of nine nursing homes due to staff and time shortages, and the remaining intervention clusters showed considerable variations in implementation fidelity and cooperation between dental assistants and nursing home staff. Participatory observations revealed that individual oral care recommendations were only completely implemented in around half of the observed participants and that more specific oral care measures (e.g., interdental cleaning) were only rarely performed. The main barriers to the intervention’s implementation were staff and time shortages, lack of interest or engagement of nursing staff, and individual resident barriers (e.g., poor health condition, uncooperative behaviour). The COVID-19 pandemic posed a major challenge during the recruitment phase, but predominantly did not affect the further course of the study, according to the participating target groups.

**Conclusion:**

Despite the overall positive perception of the intervention concept by the target groups, we found several inconsistencies in the implementation across the nursing homes of the intervention group. The barriers identified can provide indications for the future development and implementation of comparable oral health interventions in the nursing home setting.

**Trial registration:**

ClinicalTrials.gov (Trial registration number NCT04140929). Registered on 24 October 2019, https://clinicaltrials.gov/study/NCT04140929.

**Supplementary Information:**

The online version contains supplementary material available at https://doi.org/10.1186/s12877-026-08607-z .

## Background

Studies from Germany [1–4] and the international literature [5–7] indicate that the oral health of nursing home residents is often inadequate, with several oral health issues being prevalent among this population including poor dental and periodontal health, a high prevalence of mucosal problems, dental caries, tooth loss, and a compromised oral health-related quality of life. Due to complex physical and cognitive impairment, the majority of residents require substantial support in the activities of daily living, including oral hygiene. However, the delivery of adequate oral care in nursing homes (NH) is frequently impeded by various factors, such as staff shortages, time constraint, insufficient qualification or knowledge of staff or a low prioritisation of oral hygiene [8–13]. Despite known challenges and high relevance of oral health for quality of life of older people, few systematic initiatives have been taken to sustainably improve oral care in NHs. In 2010, the German Federal Dentists Association and the National Association of Statutory Health Insurance Dentists introduced the national concept ‘Oral health despite handicap and high age’ [14]. This initiative enabled contractual collaboration agreements between dentists and NHs, in order to promote structured dental care provision. As part of these contractual collaborations, residents are entitled to biannual dental examinations to assess oral, dental and maxillofacial health. The results of the examinations are documented by the dentists using a standardised form, and the NH staff receives tailored oral hygiene and dental treatment recommendations for the residents. Based on this approach, we have developed a complex intervention programme to improve the oral health of NH residents, which included six-monthly visits by dentists and was supplemented by regular visits by dental assistants (DAs). The effectiveness of the programme was investigated in a parallel-group, cluster-randomised controlled trial (cRCT) with 12 months follow-up. The primary outcome was defined as oral health-related quality of life. Other outcomes were general health-related quality of life, pain, caries experience/tooth loss, oral hygiene status, gingival inflammation and prosthetic hygiene. Further details on the methods of the main study have been published elsewhere [15]. The cRCT was accompanied by a comprehensive multimethod process evaluation and a health economic evaluation. An overview of the overall study design is presented in Additional file 1. In this paper, we report on the comprehensive multimethod process evaluation conducted alongside the trial. This process evaluation was guided by the UK Medical Research Council (MRC) guidance for process evaluations of complex interventions [16]. Our main objectives were to gain insights into the implementation process and context, as well as into the experiences of the involved target groups. The following research questions were addressed:

1. To what extent were the components of the complex intervention implemented as intended?
2. Which facilitators or barriers influenced the implementation of the intervention?
3. What were the experiences of the target groups (e.g. dentists, DAs, NH leaders, NH staff) during the course of the study?
4. Which contextual factors may have influenced the intervention and to what extent?

## Methods

### Participants of the MundZaRR trial

The MundZaRR study [15] enrolled NH residents with moderate to high levels of care dependency, classified as care levels three to five by assessors of the German Medical Advisory Service, which considers factors such as mobility, cognitive and communicative abilities, daily living organisation, and social interactions. In the German long-term care insurance system, these care levels are used to determine entitlement to care benefits and to reflect the extent of support required in daily life. Care level three to five correspond to severe to most severe impairments of independence and substantial nursing care needs [17]. Respite care recipients were excluded from the study. At the cluster level, exclusion criteria comprised existing dental cooperation agreements (according § 119b SGB V of the German social insurance). The planned sample size of *n* = 618 residents from 18 NHs was based on the effect size estimated from the feasibility study and adjusted for cluster randomisation (average 34 residents per NH; intracluster correlation = 0.05), with an anticipated dropout rate of approximately 30% due to death or severe morbidity. This target sample size could not be reached due to various constraints, most notably the SARS-CoV-2 pandemic. In total, 18 NHs with 358 participants were recruited. Nine NHs (*n* = 178 residents) were randomised to the intervention group (IG) and the other nine to the control group (CG) (*n* = 180 residents). Randomisation was performed in blocks with variable lengths [15]. Of the 358 residents who were recruited, 87 were lost to follow-up prior to baseline data collection due to reasons such as death (*n* = 45) or withdrawal (*n* = 8). This resulted in a final sample size of 271 residents. Due to the nature of cRCTs, the unit of analysis was the NH, rather than the individual resident.

### MundZaRR intervention

The intervention was developed according to the recommendations of established guidelines [18]. In the initial stages of the project, a scoping review was conducted, and an evidence map was created to provide an overview of the effectiveness of different oral care interventions [19]. This evidence map was used to inform the development of the intervention components. To determine the most suitable components for everyday oral care, interviews with dentists and NH staff (e.g. managers and nurses) were conducted to identify barriers and facilitators influencing the implementation of oral care measures, and to assess the applicability of the intervention components [20].

**Fig. 1:**
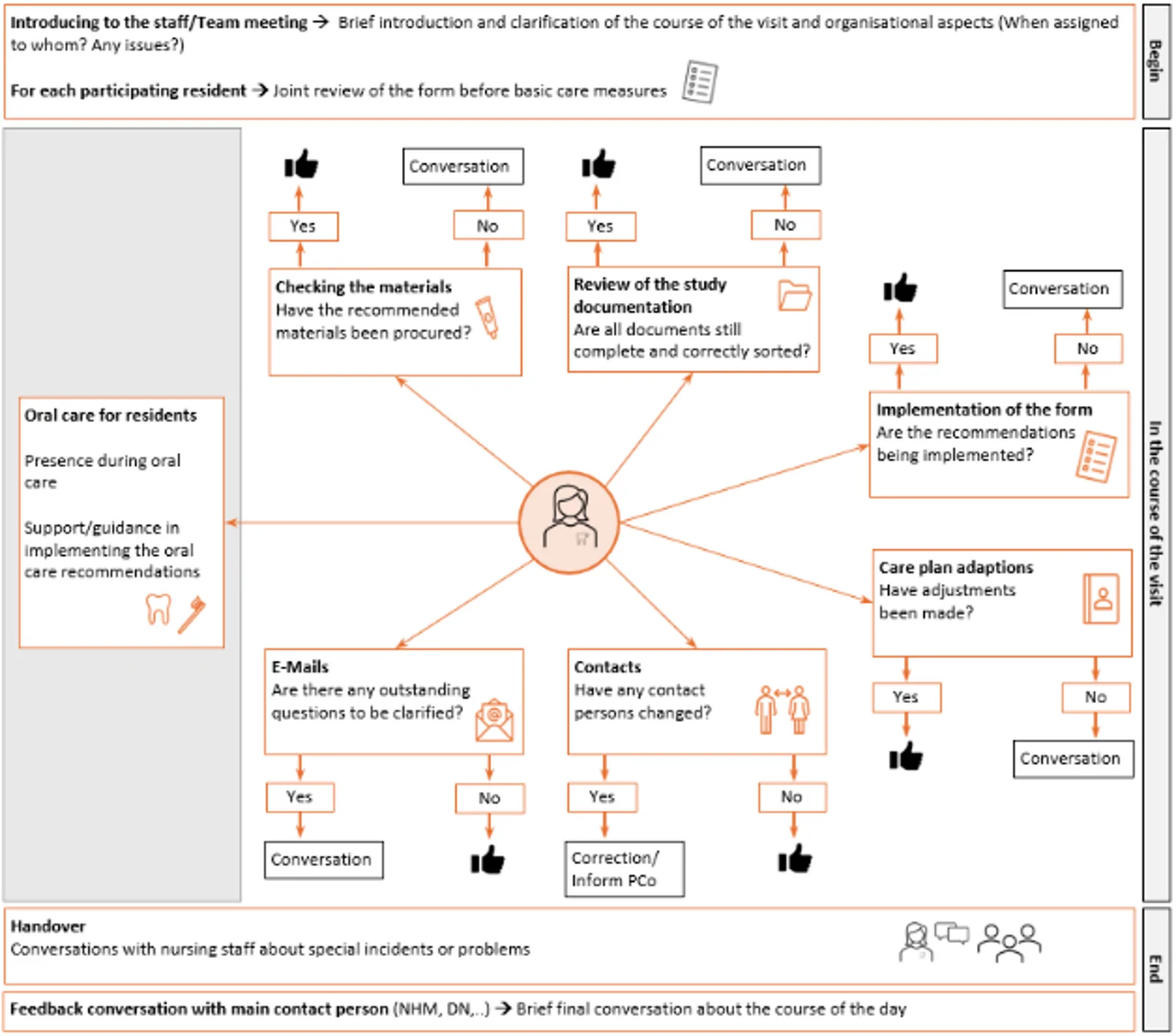
Exemplary sequence of a DA visit to the NHs. Abbreviations: DN= Director of nursing, NHM= Nursing home manager, PCo= Project coordinators

NH residents in both study groups underwent assessments by dentists at baseline, after six and after 12 months and received personalised oral hygiene and care recommendations as well as treatment recommendations. Primary and secondary outcomes were also assessed at each of these visits. The dentists utilised the standardised form, as described in the background section, to instruct and motivate the nursing staff on individualised oral care measures. In the IG, DAs then conducted regular visits to the NHs during the follow-up periods to provide ‘reinstruction’ and ‘remotivation’ for the nursing staff, based on the dentists’ initial recommendations [15]. This reinstruction included support for implementing recommended oral hygiene measures, such as guidance or advice on improving oral hygiene practices, and assistance in obtaining the necessary oral hygiene materials. Remotivation involved discussions with the nursing staff to enhance their awareness of oral hygiene and to develop tailored strategies for individual residents. Furthermore, the DAs were supposed to visit all participants at least twice at approximately 4-week intervals in the follow-up periods. An overview of the exemplary sequence of a visit by the DA to the NHs is shown in Fig. 1. In the CG, no further measures were applied. Prior to the start of the cRCT, the dentists and DAs of the IG received educational trainings. The training curriculum and manual was developed by two dentists involved in the project with extensive clinical and academic expertise in gerodontology, in collaboration with a representative of the German Society of Gerodontology. It was oriented towards this society’s continuing education curriculum. One training session was provided for both the DAs and the dentists and combined a theoretical and a practical component. The theoretical component consisted of a lecture on gerodontology and specific aspects of oral care for NH residents. The practical component involved real NH residents and consisted of repeated cycles of clinical assessment by the dentist including the completion of the standardised form and communication of the findings and oral health care plan to nursing staff for each resident. The DAs then practised interpreting the form and supporting nursing staff in implementing the communicated care plan. Each cycle was followed by joint reflection session. The DAs received further educational training on their roles and activities within the study. This online training session focused on the DAs’ tasks of ‘remotivating’ and ‘reinstructing‘ nursing staff regarding the dentist’s recommendations between assessment visits including strategies to raise nursing staff awareness, provide positive feedback and support integration of recommendations into individual care plans as well as on study logistics and the explicit scope and limits of the DA role (e.g. not training nursing staff themselves or actively intervening in the care process).

### Design

A multimethod process evaluation was conducted alongside the cRTC. The evaluation was guided by established frameworks for evaluating complex interventions [16]. We employed qualitative and quantitative methods to gain a more comprehensive understanding of the implementation process of the complex intervention and addressed multiple target groups, including dental professionals, NH managers, members of the resident council, and nursing staff.

### Setting

The MundZaRR study was conducted in NHs located in both urban (*n* = 6) and rural areas (*n* = 12) in southwestern Germany. Individual clusters were NHs varying in size from a minimum of 24 beds to a maximum of 190 beds.

### Data collection

The evaluation of educational training sessions took place before the start of the cRCT, while all subsequent data collections took place between July 2022 and October 2023. Data were collected at the start of the study (t_0_), after three (t_1_), six (t_2_) and nine months (t_3_), and at the end of the study after twelve months (t_4_).

The educational training sessions were evaluated using self-developed standardised questionnaires, which were developed based on a targeted literature review and existing questionnaires, covering the dimensions self-efficacy and general satisfaction with the training. Lecturers received one-page structured protocols in a checklist format to document the training session details (e.g. duration, number of participants) and any deviation from the training manual.

In order to document the on-site visits to the NHs and to gain a better understanding of the implementation of the intervention and its implementation fidelity, the DAs completed structured protocols after each visit. In these protocols, remotivation and reinstruction tasks were recorded as well as challenges and barriers encountered.

We also conducted open, non-participant observations to assess the acceptance and implementation of the individual oral hygiene recommendations provided by study dentists, as well as the implementation of oral care practices by nursing staff. A structured protocol guided the observations, including the documented recommendations, aspects of oral and prosthesis care, handling of care-resistant behaviour, and staff–resident interaction. The protocol was developed based on criteria and recommendations of the national expert standard on oral health promotion in nursing care [21] and additional supplementary recommendations from nursing textbooks and professional guidance documents on oral health care for care-dependent people. To standardise data collection, a detailed observation manual operationalised all observation criteria and specified concrete indicators for each observation item, including resident positioning, preparation and availability of oral hygiene materials and the systematic performance of oral care procedures. Observations took place during morning care or after breakfast in the resident’s room—either in the bathroom or at the bedside for bedridden residents—and began when staff initiated oral hygiene and ended when materials were put away or the resident left the bathroom, depending on which situation occurred first.

A document analysis was carried out to explore whether and to what extent the individual oral hygiene recommendations were integrated into NHs’ care planning and resident records. Furthermore, notes taken by the nursing staff in the study documentation were analysed regarding implemented oral care measures per residents or reasons for the non-implementation. The data were collected using a self-developed data sheet, which was pretested during the first data collection and subsequently revised.

In focus groups and individual interviews, individual experiences of relevant target groups (e.g. dentists, DAs, NH managers) as well as their perspectives on possible facilitators and barriers to the study were explored. Prior to the interviews, participants received information about the the interviewer’s role within the research project and the purpose of the interviews. Information on reasons for non-participation were not systematically collected. The interviews were performed online, by telephone or in person, according to the participants’ preferences. Only the participants and the interviewer were present during the interviews. The semi-structured interview guides were developed by the research team based on the objectives of the process evaluation and targeted literature searches in MEDLINE via PubMed combined with forward and backward citation tracking. The semi-structured interview guides were adapted for each target group (Additional file 2).

The attitudes, self-efficacy, knowledge and care practices of the nursing staff with regard to oral care were assessed in surveys using a self-developed standardised questionnaire, comprising items from validated related questionnaires [22–25]. The questionnaire was pretested with three nurses not involved in the study, resulting in a minor revision. The final version comprised 39 items in the domains attitudes (13 items), self-efficacy (11 items), knowledge (12 items), and nursing practice (3 items) related to oral care in a closed question format as well as additional questions to collect socio-demographic data.

Standardised short surveys were developed to assess structural changes in the NHs during the study period, including staffing levels and staff turnover, the implementation of concepts or standards for oral hygiene as well as of the conducting of related educational training shortly before or during the study period. These surveys were enclosed in the study documentation folders, which all NHs received before the study started. NH managers were reminded to complete the surveys by telephone or email at the respective time of data collection.

Contextual aspects were observed throughout the entire study period, in particular with focus on the course of the SARS-CoV-2 pandemic and its impact on the study. Scientific and other developments (e.g. introduction of laws and national guidelines) that may have had an impact on the intervention were also observed and documented.

Table 1 provides an overview of the target groups addressed, methods and time points of data collection, and key aspects of the evaluation. All data collection instruments are available on reasonable request.

**Table 1:** Data collection during process evaluation

Target group	Methods and measurement points	Key aspects
Lecturers of the training sessions	*Short protocols after each training session*	Duration, number of participants, deviations/adjustments from the training manual
Participants of the training sessions	*Standardised questionnaires (in IG) after the training sessions*	Satisfaction with training, self-efficacy
Dentists	*Semi-structured interviews at t*_*0*_*and t*_*4*_ *(IG: n = 1, CG: n = 1 per measurement point)*	Assessment and instruction: duration, content, acceptance, feasibility, adjustments, implementation fidelity of the oral care recommendations
Dental assistants	*Focus groups (n = 2; all dental assistants) at t*_*1*_ *and t*_*4*_	Implementation of reinstruction and remotivation, experiences
	*Structured protocol after each visit in a nursing home*	Implementation of reinstruction and remotivation, challenges
Nursing staff	*Focus groups (n = 3 in IG) at t*_*3*_	Acceptance, feasibility, adjustments
	*Survey (30% of nursing staff in IG and CG) at t*_*0*_ *and t*_*4*_	Knowledge, self-efficacy, attitudes regarding oral care and oral care practice
Nursing home managers and members of the resident council	*Semi-structured interviews (in IG) at t*_*1*_	Expectations, experiences, attitudes, acceptance
Residents and nursing staff	*Participatory observations (n = 20 in IG) at t*_*1*_	Performance of oral care, implementation of individual recommendations, interaction between care staff and residents
	*Document analyses (30% of documentation in IG and CG) at t*_*2*_	Implementation of oral care and treatment recommendations, integration of recommendations into nursing documentation, reasons for non-implementation
Structural changes on nursing home level	*Short survey at t*_*0*_, *t*_*2*_ *and t*_*4*_	Staffing levels, fluctuation, implementation of training on oral care or oral care standards
Context	*Observation during the entire study period*	Scientific and other developments that could have an impact on the intervention (guidelines, laws, etc.)

Measurement points: t_0_= baseline; t_1_ = 3 months; t_2_ = 6 months; t_3_ = 9 months; t_4_ = 12 months, Abbreviations: CG=control group; IG=intervention group

All qualitative data collections were carried out by the first author (LW), who is a research fellow at the study centre in Halle (Saale), holds a Master’s degree in Health and Nursing Science and has completed vocational training as a medical laboratory technician. LW also has comprehensive experience in conducting qualitative interviews and theoretical knowledge of carrying out non-participant observation. No prior relationship existed between LW and any of the participants of the interviews or observations.

### Sampling

At the beginning of the cRCT, all the involved NHs were informed about the process evaluation and invited to participate. Due to time and staffing constraints, one NH participated only in the data collection of the main study and therefore did not contribute to the process evaluation either.

After each educational training session, all participants were invited to take part in the evaluation and received standardised questionnaires. Lecturers were asked to fill out the structured protocols.

A convenience sample of NHs was selected for the participatory observations and document analyses, considering criteria such as the size and location of the NHs. For the participatory observations, a purposive sample of *n* = 20 residents of the IG was selected by the responsible NH staff, taking into account different levels of care dependency and need for support with oral care. The document analyses were carried out for a random sample of 30% of all residents in the IG and CG at the time of data collection.

Convenience samples were interviewed in focus groups and individual interviews: study dentists from both IG and CG; DAs, NH managers, NH staff, and members of the resident council (residents or family members nominated as representatives) from the IG. Potential participants were contacted via email or telephone by the study team. Information on reasons for non-participation were not systematically collected.

For the written survey, a 30% subsample of the nursing staff in the IG and CG was selected at t_0_ and t_4_ randomly by the contact persons responsible for the study on-site. Hence, the samples were independent at both measurement time points.

### Data analysis

All interviews were digitally recorded and subsequently transcribed in pseudonymised form by a study nurse at the study centre, University of Halle, following the transcription rules by Dresing & Pehl [26]. The transcripts were reviewed by the first author of this study (LW). For the analysis, the multi-step approach of qualitative content analysis according to Mayring [27] was applied. Initially, a set of main categories were defined deductively based on the underlying research questions of the process evaluation and the thematic domains of the interview guides, including interaction with the NH or study staff, changes observed during the course of the study, barriers and facilitators. These categories were applied during the coding of the first interviews by two independent members of the study team and were later expanded by new categories derived inductively from the interview material. Discrepancies in coding were resolved through discussion. Based on the resulting category systems, all further interviews were mainly coded by one member of the study team, and a subset of twelve interviews was checked by another. The respective category systems were iteratively discussed with two team members, during which categories were revised, expanded or supplemented, resulting in the final coding agendas. These coding agendas contained category definitions, coding rules and illustrative examples and were refined during analysis. Interview transcripts and findings were not returned to participants for comments or validation.

All quantitative data were analysed descriptively (absolute and relative frequencies, mean values, min-max). We used SPSS (version 28.0) and Microsoft Excel (Microsoft Office 2016) for quantitative data analyses. Qualitative data was analysed using MAXQDA 2022 and Microsoft Excel (Microsoft Office 2016).

## Results

### Evaluation of the educational training sessions

#### Educational training on gerodontology and practical training

Four of the five IG dentists and all four DAs participated in the training sessions. One dentist who was unable to attend the initial training session received the training at later date. The initial training lasted 210 min, the retraining 120 min. Both sessions were conducted as planned and no deviations were reported by the lecturers.

The eight participants of the initial training session evaluated the training; no evaluation data was available for the retraining. Overall, the educational training was rated positively by the dentists and DAs. All participants felt that the gerodontology content was explained comprehensibly. The dentists confirmed that they felt confident in using the standardised form, which was utilised to document individual recommendations for oral hygiene, and in applying it for the instruction of nursing staff. The DAs also stated that they were prepared for the interpretation of the form, its application, and the conduction of remotivation and reinstruction. After completing the training, the majority of participants also felt confident regarding interprofessional communication with nursing staff and appropriate handling of challenging behaviour by residents. All participants felt that the group size and comprehensibility of the content were appropriate. Two participants rated the duration of the training or the educational level as rather inappropriate.

#### Educational training for dental assistants

The educational training took place with four DAs and lasted 195 min. For pandemic reasons, the training session was conducted online; there was no other deviation from the training manual.

Three DAs evaluated the training. All areas related to the training content (e.g. background information, content and procedure of the study) and their confidence in implementing the intervention (reinstruction and remotivation measures, handling of the study materials, compliance with hygiene measures) were rated positively. They also expressed overall satisfaction with the training, stating that the duration, the educational level, and the group size were appropriate and that the contents were presented understandably.

### Implementation of the intervention

#### Implementation of remotivation und reinstruction by dental assistants

As described in the methods section, the DAs documented each NH visit using a structured protocol. Overall, 84 visits to eight NHs were protocolled. As reported in Sect. Sampling, one NH was available for the main data collection only and therefore not visited by a DA. Furthermore, one DA dropped out after a short time; her tasks were compensated for by the remaining three DAs. The DAs were supposed to visit each participant at least four times during the study. However, the realised number of visits remains unknown due to the anonymised documentation. The number of visits per NH ranged from six to 16, with a mean of 10.5 visits (standard deviation (SD) = 3.66).

**Fig. 2:**
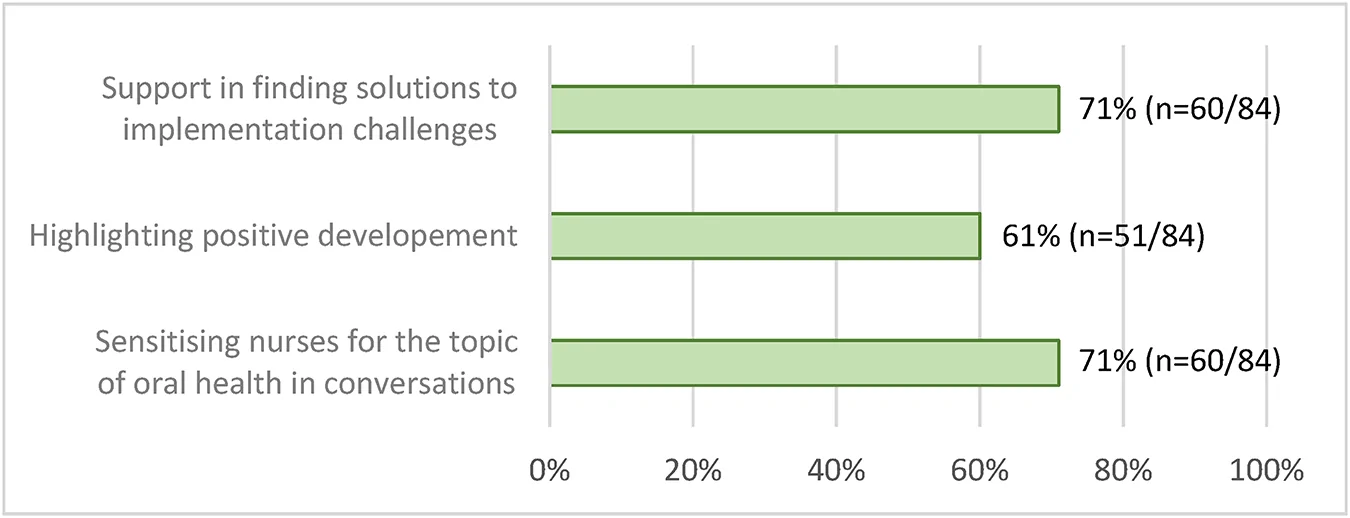
Frequency of remotivational measures

In the context of remotivation, the DAs most frequently reported engaging in conversations with nursing staff to raise their awareness about oral hygiene (71% of visits; *n* = 60/84). In an equal number of visits, the DAs were able to help the nursing staff to find solutions on how to deal with implementation challenges together. This could include providing tips for a more effective application of the measures or addressing specific professional questions raised by the staff. In 61% (*n* = 51/84) of the visits, DAs acknowledged positive development, e.g. by highlighting noticeable enhancements in residents’ oral hygiene or providing positive feedback, for instance, when recommended oral care materials were procured (Fig. 2).

61% (*n* = 51/84).

71% (*n* = 60/84).

71% (*n* = 60/84).

The most commonly reported reinstructive measure was supporting the nursing staff in the implementation or forwarding of the recommendations of the standardised form (74% of visits; *n* = 62/84). This included exploring whether all the relevant nurses were aware of the recommended oral care measures by asking whether and when the form had been discussed within the team. If needed, the DAs reminded the nurses, who received the form after the dental examination, to share its contents with colleagues, for example during handovers. In addition, the DAs offered to clarify any open questions together with the nurses. In 73% of visits (*n* = 61/84), the DAs reviewed the study documentation folders, where nurses recorded their study-related activities and implemented measures for each resident on a weekly basis. If the documentation was incomplete or inconsistent, the DAs were supposed to remind the nurses to complete the records or to assist them in doing so.

**Fig. 3:**
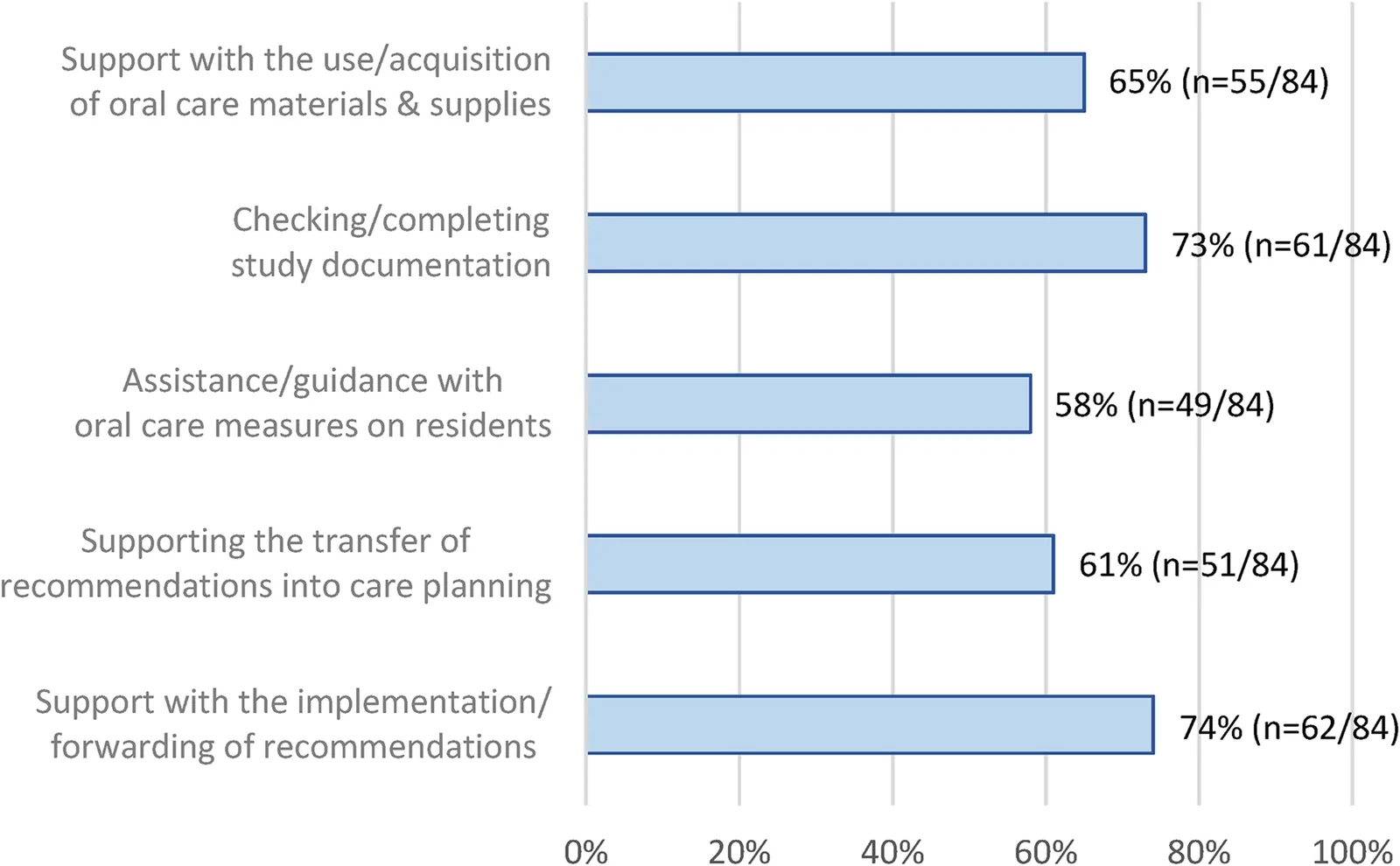
Frequency of reinstructive measures

In 65% of visits (*n* = 55/84), the DAs supported the acquisition or use of recommended oral care materials, for instance, by checking whether necessary material, such as interdental brushes or special oral care products, had been procured and were available for use. If certain items were missing, they inquired about the reasons and if necessary, helped to find solutions, for example reminding nursing staff to follow up with relatives or legal guardians responsible for purchasing the material. In 61% of visits (*n* = 51/84), the DAs reported supporting the incorporation of oral care recommendations into the NH’s care plan by e.g. discussing necessary adaptations with the nursing staff. The least frequently reported activity was the guidance and assistance of nursing staff in implementing oral care measures on the residents, which occurred during only 58% of visits (*n* = 49/84). This may have included, for example, providing assistance when residents refused to open their mouth, assisting with the cleaning of removable dentures, or helping nursing staff who experienced difficulties in cleaning teeth and oral mucosa (Fig. 3).

In a total of 19 visits to five NHs, no resident visits were conducted. Reasons were refusal by nursing staff due to inconvenient visiting times, as well as a lack of time or staff resources. In some cases, DAs visited NHs solely to check study documentation folders.

#### Delivery of oral care and implementation of individual oral hygiene recommendations

In three NHs of the IG, the delivery of oral care and individual recommendations by nursing staff was assessed through direct observation (*n* = 20). These observations lasted approximately six minutes per resident (min = 3, max = 8 min). The observed residents were predominantly female (85%) and on average 81.5 years old (min = 64, max = 94 years). The nursing staff accompanied were also mostly female (88%) and on average 44.5 years old (min = 22, max = 67 years). Other descriptive data are displayed in Additional file 3.

Overall, oral care was mostly carried out correctly or instructed appropriately by the nursing staff. However, in some cases, the oral care provided was rather inappropriate. For example, the brushing time was sometimes too short (approximately one minute) or the oral care provision did not follow the standard procedures of oral care. Furthermore, a lack of instruction by the nursing staff or inadequate hygiene measures for self or resident protection (e.g. not wearing disposable gloves or hand desinfection) were observed. The oral hygiene recommendations were fully implemented in less than half of the situations observed (45%; *n* = 9/20). However, the recommendations were considerably more often fully implemented in one NH than in the other two. More specific measures, such as tongue or oral mucosa cleaning, were only carried out in individual cases, and interdental space cleaning was conducted in one NH only. The main barrier was the lack of necessary supplies. Other general challenges in the implementation of oral care and individual recommendations were e.g. care-resistant behaviour or residents’ physical impairment.

#### Inclusion of recommendations in care planning and implementation of dental recommendations

Document analyses was carried out in six NHs (IG: *n* = 3, CG: *n* = 3). Overall, 89 resident records (IG: *n* = 43, CG: *n* = 46) were screened. In the IG, oral hygiene recommendations were completely incorporated in the NH’s care planning for 14% (*n* = 6/42) of the residents, at least partially for half of the residents (50%; *n* = 21/42) and not at all for 36% (*n* = 15/42). This was comparable in the CG (9% (*n* = 4/44) completely, 52% (*n* = 23/44) partially and 39% (*n* = 17/44) not at all).

The IG nursing documentation revealed various reasons for the non-implementation of oral hygiene measures and recommendations. These included a lack of cooperation or compliance of residents, e.g. when oral hygiene conducted by nursing staff was refused or more specific measures such as tongue cleaning were not accepted. Financial and organisational barriers were also apparent. These included, for example, that supplies for oral or denture cleaning were not procured by relatives, financial resources for the acquisition of materials were lacking or the nursing staff had insufficient time available. Individual risks for the residents (e.g. aspiration risk) were further reasons for the non-implementation. In the CG, no reasons for non-implementation could be assessed due to a lack of documentation.

Overall, the IG residents received more treatment recommendations compared to the CG residents (*n* = 70 vs. *n* = 42 recommendations in total). On average, IG residents received 1.67 (SD = 1.36) treatment recommendations, whereas CG residents received 0.98 (SD = 0.99) recommendations per resident (median: 2 vs. 1). The most frequent recommendations in the IG were dental fillings (*n* = 19/70) and treatment of the dentures (*n* = 16/70), while in the CG, treatment of the dentures (*n* = 15/42) was most frequently recommended, followed by treatment of the gingiva or oral mucosa (*n* = 7/42). There was no need for treatment for 30% of the residents (*n* = 13/43) in the IG and 39% of the residents in the CG (*n* = 18/46). The actual implementation of the dentist’s treatment recommendations was found for six (20%) residents in the IG and only two residents in the CG (7%). In addition, further measures were documented for ten residents in the IG (23%), mainly involving discussions with relatives regarding the dental recommendations, some of which were rejected by the relatives.

### Knowledge, attitudes, self-efficacy and care practice with regard to oral hygiene in the nursing homes

A total of 103 nurses participated in the written survey at t_0_ (IG: *n* = 50, CG: *n* = 53) and 108 at t_4_ (IG: *n* = 44, CG: *n* = 64). The overall response rates were 42% (*n* = 108/260) and 47% (*n* = 115/245), respectively. Response rates by study group were 38% (*n* = 56/146) in the IG and 46% (*n* = 52/114) in the CG at baseline, and 48% (*n* = 62/129) and 46% (*n* = 53/116), respectively, at t_4_. At t_0_, *n* = 5 questionnaires and at t_4_*n* = 7 questionnaires had been completed by trainees or other NH staff and were therefore excluded from the analysis. Characteristics of the participating nursing staff can be found in Additional file 4. The detailed results of the individual domains are displayed in Additional file 3.

#### Attitude towards oral hygiene

Both, nurses in the IG and nurses in the CG showed a predominately positive attitude towards oral hygiene at both survey times, whereby the participants in the CG tended to have a slightly more positive attitude towards oral hygiene at the end of the study compared to the IG (Figure S2 and S3 in Additional file 5). Almost all nurses considered oral care to be an important task (IG: 96% at t_0_ and 95% at t_4_ and CG: 100% at both survey times) and were predominantly convinced of the positive effects of appropriate oral care. However, nurses also revealed negative attitudes. This concerned e.g. denture care, which was considered less problematic than the care of natural teeth by 65% and 64% of the IG nurses at t_0_ and t_4_, respectively, and by 62% and 67% of the CG nurses. In addition, 57% (t_0_) and 52% (t_4_) of the IG nurses were convinced that residents would request assistance with oral care independently. In the CG, 46% (t_0_) and 59% (t_4_) of the nurses thought that cleaning of teeth and dentures should not be left to residents, even if possible.

#### Self-efficacy

Self-efficacy with regard to oral care was also high in both groups at both survey times (Figure S4 and S5 in Additional file 5). The majority of nurses in the IG and CG reported feeling confident in conducting both regular (IG: 88% at t_0_ and 91% at t_4_; CG: 92% at t_0_ and 97% at t_4_) as well as specialised oral care and specialised oral care (IG: 82% at t_0_ and 88% at t_4_; CG: 79% at t_0_ and 89% t_4_). The nurses were convinced that they can make a valuable contribution to maintaining the oral health of their residents. In addition, integrating oral care into everyday work routines was not seen as a problem by most of the respondents (IG: 88% at t_0_ and 91% at t_4_; CG: 96% at t_0_ and 88% at t_4_). The greatest uncertainty was found in conducting oral care for residents with dementia, which was perceived as particularly challenging by 74% of the IG nurses at t_0_ and 77% at t_4_, and by 70% of the CG nurses at t_0_ and 63% at t_4_.

#### Knowledge

Overall, both groups had a good level of knowledge regarding oral hygiene. However, CG nurses tended to have a slightly higher level of knowledge (Figure S6 in Additional file 5).

Almost all respondents in the IG and CG knew at the beginning and end of the study that bacterial plaque in the mouth can cause infections of the gingiva (IG: 96% at t_0_ and 91% at t_4_; CG: 96% at t_0_ and 95% at t_4_) and that brushing with a toothbrush and toothpaste is the best way to remove plaque and deposits (IG: 90% at t_0_ and 98% at t_4_; CG: 94% at t_0_ and 92% at t_4_). However, some knowledge gaps remained. For instance, only 13% of the IG nurses at t_0_ and 16% at t_4_, and 15% of the CG nurses at t_0_ and 7% at t_4_, correctly identified that frequent sips of water are not always the best way to treat dry mouth. Likewise, only 38% of the IG nurses at both t_0_ and t_4_, and 50% of the CG nurses at t_0_ and 50% at t_4_, correctly knew that dentures do not necessarily have to be removed overnight.

#### Oral care practice

At the end of the study, a substantially higher proportion of nurses in the IG compared to the CG stated that they were able to provide adequate oral care twice per shift (Figure S7 in Additional file 5). Overall, 86% of the IG nurses at t_0_ and 73% at t_4_, and 62% of the CG nurses at t_0_ and 63% at t_4_, reported having up to five minutes available for oral care per resident. However, the CG respondents tended to have slightly more time available to perform oral hygiene per resident (Figure S8 in Additional file 5) but were more often not able to conduct oral hygiene for a resident at all compared to the IG respondents (Figure S9 in Additional file 5).

### Experiences of the individual target groups

We performed 23 individual and three focus group interviews with 24 participants. An overview of the sample characteristics can be found in Additional file 6.

#### Experiences of the study dentists

Seven dentists (IG: *n* = 4, CG: *n* = 3) participated in the t_0_ interviews and six (IG: *n* = 3, CG: *n* = 3) in the t_4_ interviews. The average duration of the interviews was 30 min (min = 18 min, max = 43 min). The dentists were mostly male (*n* = 6) and the mean age was 56.7 years (min = 40, max = 67).

Most dentists were able to discuss the forms with individual oral care recommendations with one or two nursing staff members during data collection visits. In some cases, the instruction was limited to the NH managers or nursing staff on duty in the residential departments, or was not possible at all. In the IG, each dentist was additionally accompanied by a dental assistant (DA) during data collection at baseline and after six months. The individual recommendation forms were usually discussed with the DA to ensure shared understanding of the proposed measures and any indicated dental treatments.

Almost all dentists reported that their recommendations were hardly or not at all implemented. Accordingly, they observed almost no changes in the oral health of the residents or oral care practices.

The dentists perceived the interest and commitment of the nursing staff as decisive influencing factors:

*‘But these visits and this cooperation […] of the DAs*,* […] I actually thought that was a good idea. But only if the nursing staff go along with it. […] Because in [NH 6] she [the DA] tried to get involved*,* but the staff resigned. She couldn’t talk to the people at all. […] And the nursing staff*,* we wrote the notes and they didn’t do anything. The whole [study] folder was not processed in any way.’ (Dentist 03_IG)*.

Many dentists also saw a shortage of staff and time as a barrier, particularly for the implementation of more specialised oral hygiene measures:

*‘Well*,* I can’t expect the nursing staff to help the resident twice a day with interdental brushing*,* because there simply isn’t enough time*,* to be honest.’ (Dentist 07_CG)*.

As some NHs had to rely on using temporary staff, there was also a lack of knowledge or qualification for implementing the recommendations in some cases.

At the individual level, the poor health condition of some residents also restricted implementation:

*‘The form […] as a guide for the nursing staff on what you should do*,* what you can do […] but that’s always a bit difficult depending on the patient’s health condition. I can’t write down chlorhexidine or say that he [the resident] should rinse in the morning for a few days to get rid of the gingivitis or whatever*,* if he drinks litres of it.’ (Dentist 3_IG)*.

The dentists identified various aspects they believed were necessary to effect changes in the residents’ oral health. The importance of educational training for nursing staff was frequently highlighted as a means of enhancing the knowledge and expertise of staff in geriatric dentistry. Other approaches included, for example, more frequent and regular visits by DAs to enable them to foster a relationship with the residents and the NHs, as well as providing better equipment for examining the residents.

#### Experiences of the dental assistants

The two focus group interviews with the DAs (*n* = 3) lasted 65 and 69 min. All DAs were female and aged 26 to 31 years (mean = 28.3).

The DAs’ experiences regarding the implementation of the intervention were strongly influenced by the quality of cooperation with the NH staff, which varied considerably between, and sometimes within, NHs. The DAs reported that the cooperation in some NHs was very collegial, while in other NHs they felt unwelcome and were unable to implement their activities as planned.

Similarly, the implementation of the oral hygiene recommendations varied considerably between NHs. While some NHs largely adopted the measures — e.g., acquiring supplies or arranging dental appointments — others showed little motivation to adjust standard care routines and followed the recommendations only in acute cases. A lack of interest and sustained commitment from nursing staff was frequently cited as a major barrier. The DAs reported difficulties in securing sustained cooperation from nursing staff, despite initial agreements to implement necessary measures:

*‘[…] when I said that this or that should perhaps be improved or something*,* I was always told: »Yes*,* of course*,* it will be done.« But you couldn’t rely on that. […] I met with a lot of disinterest.’ (DA_03)*.

Another key barrier identified was the persistent shortage of staff and time within NHs, which limited opportunities for collaboration between DAs and nursing staff. The high workload in daily care routines often left little space for oral hygiene measures, making it difficult to ensure continuity and integration of the recommendations into standard care practices, as well as for DAs to implement the envisaged remotivational or reinstructive activities.

Beyond staff-related barriers, challenges also emerged from residents or their relatives who questioned the necessity of specific recommendations, e.g. regarding dental treatments or the acquisition of specific oral care supplies. Furthermore, a poor general health condition of some residents — such as severe frailty or cognitive impairments — posed practical limitations to intervention uptake.

Despite these challenges, DAs emphasised that in NHs where engagement was higher, the intervention was more successfully implemented. The presence of motivated staff members and supportive leadership appeared to be crucial facilitators.

As proposed by the study dentist, the DAs also suggested that specific educational training for nursing staff would have been a necessary addition to the intervention programme.

#### Experiences of the nursing home managers

The interviews with NH managers of the IG (*n* = 7) lasted an average of 25 min (min = 21 min, max = 33 min). All NH managers were female, on average 40.9 years old (min = 33, max = 57) and had two to 15 years’ experience in leading positions (mean = 8.0).

In the interviews with NH managers, the importance of oral care in their NHs was discussed. In general, they considered oral care a lower priority in the daily care routines compared to other care-relevant tasks. Two managers rated the importance in their facility as high, but at the same time emphasised that it is often more difficult to carry out oral hygiene for residents with a higher level of care dependency.

*‘‘The significance [of oral care] is actually very high […]. However*,* it is often somewhat neglected in residents with higher levels of care dependency. Or you have fewer ideas about what you could do for oral care.” (Nursing home manager_04)*.

The cooperation with dentists and DAs was described as positive, pleasant and uncomplicated by the majority of NH managers. Some participants noted improvements in oral hygiene among relatively autonomous residents who paid more attention to their own oral care based on the dentists’ recommendations. Additionally, some NH managers mentioned the organisation and regular use of new tools, such as interdental brushes and three-headed toothbrushes, and that they had noticed an increased attention to oral hygiene among their staff. Despite these improvements, participants highlighted several challenges in implementing the intervention. Accessing dental care was particularly difficult for immobile or multimorbid residents.

*‘[.] and making the effort*,* especially for residents with care level 3 or higher*,* usually means that they either need to be transported lying down or in a wheelchair. The effort required for a dentist’s appointment is enormous. Usually*,* an accompanying person has to be provided to help with the whole thing*,* because the driver only takes the person to the entrance*,* but the practice may still be on the third floor. Someone has to wait because the resident may not be able to express themselves – very difficult.” (Nursing home manager _01)*.

Other NH managers reported that shortage of staff and time often resulted in minimal oral care activities or discussions with relatives not taking place. Almost all participants felt the rejection of oral care measures by residents to be a challenge. This was partly attributed to cognitive impairment, but also due to the low personal value placed on oral care by some residents.

The commitment of the managers, often driven by a positive attitude towards nursing research and a desire to contribute to the project’s success, proved to be a significant facilitator. Most participants hoped that participating in the study would raise the awareness of oral hygiene and the recognition of its relevance in everyday care among their nursing staff. From the participants’ point of view, such an intervention programme should be supplemented by the provision of materials for oral care (e.g., samples of special toothpastes) free of charge and further educational training by dentists on the topic of oral health. Three of the participants expressed the wish for regular visits by a dentist to the NHs.

*‘I would wish that more dentists would agree to provide care in nursing homes*,* so that oral health might gain a higher priority*,* just like general practitioner care.” (Nursing home manager _04)*.

#### Experiences of the nursing staff

A focus group interview with four participants (duration: 42 min) and an individual interview (duration: 31 min) were conducted. Four of five interviewed nurses were female and mean age was 34.6 years (min = 28, max = 42). The nursing staff came from two NHs of the IG.

All participants perceived the cooperation with the dentists as positive, but their experiences with the DAs varied. For instance, the participants of the focus group shared rather negative experiences, stating that they found the activities of the DA rather unhelpful and perceived the visits as a form of control. One nurse explained that she felt the instructions and advice given by the DA on denture cleaning as a negation of her own qualifications.

*‘We all finished our vocational training ten years ago*,* we’ve been cleaning dentures for ten years*,* we’ve also cleaned our own teeth*,* so I don’t need anyone to stand next to me and watch how I hold the denture in my hand and how I clean it.’ (Nurse_04)*.

In contrast, during the individual interview the participant described a friendly and respectful cooperation, and found the review of the study documentation and the associated reminder of outstanding dental appointments or treatments particularly helpful. No significant changes were noted regarding the residents’ oral hygiene or general oral care practice, and oral care was largely continued in the same way as before participating in the study. The focus group participants felt that the dental recommendations did not contain anything new and were mostly insufficient to achieve changes in oral hygiene. However, they reported changes in their own behaviour, noting increased attention to oral care since the start of the study, although this had not been uniformly adopted by all staff members. Overall, the participants had anticipated greater benefits from participating in the study, such as on-site treatments by dentists, more extensive advising activities by the DAs, or a more substantial increase in knowledge.

The nursing staff predominantly identified the same barriers as the other target groups such as staff and time constraints, but they also highlighted the rejection or lack of support from residents’ relatives regarding oral hygiene measures or treatment recommendations as a relevant barrier. As also described by the DAs, some relatives expressed reservations regarding the recommended oral health measures, especially when residents were already in a worse general condition. This often put the staff in a difficult mediating role, which required additional time and communication effort.

In addition, the nursing staff pointed out that some treatment recommendations provided by the dentists were not supported or implemented by the residents’ regular dentists:

*‘Above all*,* the other dentist was quite angry and upset about having to take any measures now because another dentist said so. So that didn’t work out so well either.’ (Nurse_03)*.

An overview about all barriers mentioned by the different target groups can be found in Table 2. The participants expressed a desire for regular visits by dentists to the NHs, and partly more topic-specific training (e.g. on the consequences and clinical pictures of poor oral hygiene).

**Table 2:** Overview of the barriers mentioned by the target groups

Barriers	Reported by…				Quotes
	Dentists (*n* = 7)	DA (*n* = 3)	NHM (*n* = 7)	Nurses (*n* = 5)	
**Nursing home level**					
*Lacking interest or engagement of NH staff*	x	x	x	x	*‘I would say that all the recommendations made by the dentist are not implemented unless the patient is really in pain and no longer wanted pain*,* then maybe something would be done.’ (DA02)*
*Shortage of staff and time*	x	x	x	x	*‘I think it’s simply a lack of staff and time. So if you really realise what a nurse has to do every day*,* it’s very*,* very much. (D07_CG)*
*Lacking knowledge or qualification*	x	x			*‘Of course*,* some of the nurses are only there on a temporary or emergency basis and they have no idea about it [oral care].’ (D02_IG)*
*Lacking prioritisation of oral care*	x	x	x		*‘For the nurses*,* oral hygiene is one element of their care work and it is not necessarily a priority for them.’ (D06_IG)*
*Lacking communication*		x		x	*‘[…] but at the other NH*,* the staff didn’t talk to each other*,* they didn’t convey anything that we discussed. At the second appointment when I was there with a new nurse*,* she wasn’t informed at all*,* she didn’t know anything.’ (DA02)*
**Resident level**					
*Poor general condition*	x	x	x	x	*‘For one resident*,* we had the recommendation on the form to brush [teeth] twice a day. At some point*,* however*,* this no longer worked for him. Ultimately*,* he also had a high aspiration risk and so it’s logical to say ok*,* this can’t be implemented.’ (DA03)*
*Rejection by residents*	x	x	x		
Low personal value placed on oral care					*‘We also have this clientele*,* this generation*,* who do not attach any particular importance to well-maintained and healthy teeth and dentures. This is rather less important for most*,* not all*,* but most.’ (NHM07)*
Care-resistant or uncooperative behaviour					*‘This is not just the case in [NH 18] […]*,* but there are residents with a catastrophic oral hygiene condition*,* where it is very difficult to communicate anything. You also notice that the nursing staff is aware of this*,* but oral hygiene is hardly or not at all feasible because the residents do not necessarily cooperate.’ (D06_IG)* *‘[…] patients with dementia*,* you don’t always manage to get them to open their mouths. For example*,* I had a patient with severe dementia and during all my visits - I think I’d been to the NH 15 times - I managed to watch them perform oral hygiene on her for the first time during my last visit because she never allowed it before.’ (DA01)*
*Rejection or lack of cooperation by residents’ representatives/ relatives*	x	x	x	x	*‘Some things also fail*,* for example*,* because a relative says no*,* this and that should not be done.’ (DA03)* *‘For example*,* there was a patient who actually needed a new prosthesis because it hardly fitted properly anymore and the representative said: No*,* as long as it still fits somehow*,* then it should stay that way.’ (DA01)*
*Lacking financial resources and supplies*		x	x		*‘Some things the dentist said they [NHs] didn’t do because the patients couldn’t afford it and the health insurance wouldn’t pay for it.’ (DA02)*
**Dental care provision**					
No or difficult access to dental care	x	x	x	x	
*Difficult access due to physical or cognitive impairments of residents*					*‘[…] and with some people from care level 3 onwards, it’s no longer possible to take them there [to the dentist]. This is due to physical limitations, behavioural issues, the effort required, or the non-accessibility of dental practices – so many factors come together, and as a result, this issue simply gets pushed aside.’ (NHM06)*
Lack of willingness or availability of dentists for on-site dental care					*The problem is that dentists in our area don''t want to make on-site visits. We have this problem with general practitioners and it''s now spreading to dentists too, and it''s going to get even more difficult.’ (NHM_04)*
Rejection by treating dentists	x			x	*‘We were told that a tooth had to be removed or this and that had to be done. Then we called the NH’s dentist and he said: I''m sorry, the woman is over 90 years old, why should I treat her now if she''s not in any pain.’ (N04)*

Abbreviations: CG=control group, D=dentist, DA=dental assistants, IG= intervention group, N=nurse, NH=nursing home, NHM=nursing home managers

#### Experiences of the members of the resident council

Two individual interviews lasting 11 and 16 min were conducted with members of the resident council. Both participants were male, 68 and 84 years old, had lived in the NH for three and ten years, and had been assigned a care level of 2 and 3, respectively.

Since neither of the interviewees had participated in the study, they were unable to provide any personal experiences regarding the intervention. Furthermore, no insights or feedback from other residents were reported. Both participants were generally satisfied with the oral care provision in their NHs. At the time of the interview, both received support from relatives, e.g., in making appointments with dentists or procuring oral hygiene supplies. One participant stated that he carried out his oral care independently, while the other participant received some support from nursing staff. Both participants placed a high personal value on oral care.

### Structural changes in the nursing homes

Eight NHs (IG: *n* = 5, CG: *n* = 3) returned the questionnaires at all three survey times. Four NHs (IG: *n* = 2, CG: *n* = 2) provided incomplete or contradictory data and five other (IG: *n* = 1, CG: *n* = 4) did not participate in the survey.

No substantial differences were found between IG and CG NHs regarding staffing levels and turnover. However, three IG NHs experienced leadership turnover in positions as directors of nursing and ward managers, while only one NH of the CG reported a leadership change.

In the period of up to three months before the start of the study, two IG NHs had completed the educational training courses on oral hygiene. In one of these NHs, all nurses were trained on a new national guideline for the promotion of oral hygiene in care [21]; the guideline was implemented during the study period. In the second NH, online training was provided, however, no further detailed information was available on the content and number of participant. Another IG NH planned training on the national guideline at the end of the study, thus, follow-up data was missing. Two further IG NH reported at t_4_ that a standard on oral hygiene had been developed in the past six months and that implementation should begin after the end of the study. In contrast, no CG NHs reported educational training or standard implementation before or during the study period.

### Contextual factors

The SARS-CoV-2 pandemic was identified as a relevant contextual factor. Already during the recruitment phase of the MundZaRR study, the pandemic posed major challenges, which led to temporary interruptions in the recruitment process. Subsequently, additional or new NHs and residents had to be recruited. A detailed report on the challenges and barriers of the recruitment will be reported elsewhere. At the beginning of the cRCT, some of the appointments for t_0_ data collection had to be postponed several times, as visits to NHs were not permitted due to SARS-CoV-2 outbreaks. This resulted in a delayed start of the study in some NHs, up to six months in one NH. In the interviews, however, the dentists, DAs and NH managers predominantly reported that the pandemic no longer had a significant impact on the implementation of the study and that the visits to the NHs could be conducted as planned.

Additionally, in 2021, the first version of the national guideline for the promotion of oral health in care that provides evidence-based recommendations to standardise the quality of care and implement best practices, was published in Germany [21]. As described in the section above, two NHs of the IG reported the implementation of the expert standard during the study period.

## Discussion

Our process evaluation aimed to investigate the implementation of the complex MundZaRR oral health care intervention delivered to nursing staff and residents. Notably, the sample size of the MundZaRR study was much lower than calculated and the effect evaluation revealed no significant improvement in residents’ oral health-related quality of life. Against this background, the process evaluation provides additional insights into how the intervention was implemented in practice, the experiences of the involved target groups and what contextual factors might have influenced the trial.

The educational training sessions for dentists and DAs were largely carried out as planned and were evaluated positively by the participants, suggesting that the educational component was well-received by both dentists and DAs, preparing them well for their role in the intervention program. Both target groups stated that they felt confident regarding their planned study activities.

Regarding the intervention’s implementation, we found several inconsistencies across the nine NHs in the IG. Overall, the DAs were able to perform most of the activities envisaged by remotivation and reinstruction, but the majority of visits focused on problem-solving, discussions with nursing staff to raise their awareness of oral hygiene, and promoting the implementation or forwarding of the standardised form. In contrast, the frequency of direct support for oral care with residents was rather low. In one NH, the intervention could not be implemented at all. Inconsistencies in the implementation of the intervention were mostly related to shortages in staff and time within the NHs, which may have negatively affected the impact of the intervention. Depending on the resources available in the NHs, the DAs had to adapt the implementation of remotivation and reinstruction individually, which caused variations in the frequency of the activities in each NH and likewise the dose and reach of the intervention.

During the direct observations conducted in selected NHs of the IG, the appropriate provision of oral care was observed in the majority of cases. However, the individual dental recommendations were only completely implemented in approximately half of the cases observed. Likewise, the dental recommendations were mostly only partially or not at all integrated into the NHs’ care planning indicating that not all nurses were aware of the individual oral care recommendations, and reflecting a potential gap in communication or dissemination within the NHs. This could also have limited the reach of the intervention, as fewer residents received the recommended oral care measures.

Further, the process evaluation found predominantly positive attitudes and a high level of self-efficacy towards oral hygiene, as well as a good level of knowledge about oral care among both the IG and CG nurses. Although the MundZaRR intervention did not specifically aim to improve nurses’ knowledge, attitudes or self-efficacy with regard to oral care, this could have been an expectable consequence of the regular visits by the DAs in the IG. Nonetheless, we did not find any substantial differences between the two groups or over time in any of the domains surveyed. In terms of oral health care practices, most of the nurses in both groups reported having zero to five minutes for each resident to perform oral care, which may be insufficient to maintain or improve the oral health of NH residents. An interventional pilot study conducted in the USA found that certified nursing assistants required on average 6.7 min to provide adequate oral care covering brushing, flossing, and oral tissue cleaning [28]. However, as shown in two systematic reviews, lack of time is one of the most frequently reported barriers in providing adequate oral care to NH residents [13, 29].

In the focus group and individual interviews, both the dentists and the DAs reported considerable discrepancies in their cooperation with the NHs and the associated nursing staff. While the cooperation in some NHs was rather collegial, in other NHs there was only little support and acceptance of the study. The interviewees attributed these discrepancies to a lack of interest or commitment of some NH managers, as well as the nursing staff. On the other hand, a high commitment and positive attitude of NH managers where described as important facilitators of the study. Previous research on the implementation of complex interventions in NH settings already demonstrated that managerial support plays a decisive role towards success of interventions within NHs and is critical for the continuity of intervention delivery [30–32].

If nursing staff do not perceive the intervention as relevant or hold negative attitudes towards it, they are unlikely to continue engaging with it [33, 34]. In our study, the nurses in the focus group were rather dissatisfied with the intervention measures and would have liked to see more benefits from participating in the study. Furthermore, when the staff is confronted with time constraints and a high workload, everyday activities take precedence and study-related tasks tend to be neglected [33]. The prevailing shortage of staff and the resulting time constraints were also reported by the interviewees as main barriers to the overall implementation of the intervention and the extent to which the individual oral hygiene and care recommendations were implemented. These perceptions, however, could not be compared with objective data on staffing levels and staff turnover, as the short surveys conducted in the NHs contained a substantial amount of missing data. Nevertheless, the challenges of time constraints and staff shortages are not only barriers that specifically relate to the implementation of oral care interventions, but also to the implementation of interventions in the NH setting in general [33, 35, 36].

In addition, NH managers often found it difficult to realise the dental treatment recommendations, as access to dental care and transportation to treating dentist is particularly challenging for multimorbid and immobile residents. At the same time, interviewees reported that there is often a lack of dentists who are available to provide dental care to residents on-site, which makes it even more difficult to provide needs-orientated care. These findings align with earlier published literature [20, 37, 38]. In our health economic analysis [39], however, we observed an increased uptake of dental care services in the IG, which might also be related to the support provided by the DAs, e.g. in organising dental appointments or sensitising the NH staff for the need of dental treatments. Another significant obstacle of the intervention was refusal of oral hygiene and treatment recommendations by the residents themselves, which is, again, a common challenge in the provision of oral care in NHs [9, 13, 20, 40]. This refusal was associated to residents’ cognitive impairments, care-resistant or uncooperative behaviours or a low personal value placed on oral care.

In the light of these challenges, the importance of adequately informed and trained staff becomes apparent. The DAs, dentists, and NH managers emphasised the need for educational training for nursing staff as an approach to optimising the intervention. Several other studies investigated educational interventions addressing nursing staff [22, 41–43] while the MundZaRR intervention did not comprise such an educational component. This may be considered as additional component for future programmes to increase nursing staff knowledge and confidence in carrying out adequate oral care, e.g. in managing care-resistant behaviour, and raise awareness of the relevance of this aspect of care. However, a Cochrane Review found no evidence for a clear benefit of educational training in improving the oral health of NH residents [44]. Other suggestions from the participants for further optimisation of the intervention included more frequent visits by the DAs and increased hands-on activities, such as providing or assisting with oral care for residents or performing prophylactic measures. Such approaches have already been investigated in other studies and showed more promising results [45–47].

Furthermore, we found two contextual factors that influenced our study. First, our study was conducted during the SARS-CoV-2 pandemic, which has imposed additional workload and major organisational challenges on NHs [48–51]. Although the interviewees in our study predominantly did not report any negative impact of the pandemic on the conduct of the study, we observed that the pandemic negatively affected the interest of NH in participating in the study and the availability of staff during the study. Second, two NHs of the IG organised training courses on the national guideline shortly before the start of the study or during the study period and another two NHs of the IG had developed an oral care standard during the intervention period. The implementation of the national and other oral care guidelines might have caused a change in the awareness of the topic of oral health and increased the motivation in these NHs [52].

### Strengths and limitations

This study contributes to the still limited body of literature on process evaluations of oral health interventions in NHs. It offers valuable insights into the specific barriers and obstacles encountered during the implementation of the MundZaRR intervention. Our process evaluation was guided by established theoretical frameworks, ensuring a systematic and comprehensive approach. We employed a multimethod design incorporating both qualitative and quantitative methods that enabled the inclusion of diverse interest-holder perspectives, such as dentists, DAs and NH staff within the study. Furthermore, participation rates in the interviews with management staff, dentists and DAs were high, supporting the robustness of the qualitative findings.

However, there are some limitations. Since participation in the process evaluation and individual data collection components was voluntary, the findings may primarily reflect the perspectives and experiences from NHs and individuals who were particularly motivated or engaged. As a result, certain relevant perspectives may not be fully represented. The protocols of the DAs documented the residents’ visits anonymously; consequently, the information could not be traced back to individual residents. Due to the highly structured nature of the protocols, only a rather broad overview of the remotivation and reinstruction process could be obtained. We did not conduct a more in-depth analysis of high- and low-performing NHs as part of our process evaluation, which might have provided a more detailed exploration of the complex interactions within the clusters and the implementation process over time. A further limitation relates to the restricted scope of training evaluation. In line with Kirkpatrick’s framework [53], our assessment primarily covered participants’ reactions (Level 1), while higher levels including knowledge transfer and competency development (Level 2) or behavior change (Level 3) in practice were not systematically assessed, which limits conclusions regarding actual skill acquisition of the DAs. Likewise, no standardised pre-post competency assessment was conducted and the training evaluation questionnaires were not subjected to formal psychometric evaluation. Consequently, it remains unclear whether the DAs achieved a comparable level of competency following the training and conclusions regarding actual skill acquisition beyond self-perceived competence and knowledge are therefore limited.

In the interviews, participation of nursing staff and members of the resident council was low. Therefore, it is conceivable that other participants might have contributed additional aspects or perspectives to the analysis, which is why data saturation was probably not achieved for these target groups. No interviews were conducted with participating residents, so their personal views remain unknown. The observations were conducted openly, which may have introduced observer bias, as real-life conditions could have been altered. Moreover, they were limited to a small sample, which may not fully reflect the range of practice across all NHs. The document analysis revealed strong differences in the quality of internal nursing documentation between NHs, which is why non-documentation of oral hygiene or treatment measures cannot be equated with a non-implementation, which in turn makes the comparability of the results more difficult. The written surveys relied on independent samples and therefore limits the comparability between the study groups and the different measurement points. Furthermore, self-reported attitudes towards oral hygiene may have been influenced by social desirability bias, potentially resulting in more positive responses that do not necessarily translate into actual oral care practices. Self-efficacy ratings are likewise subject to overconfidence and biased self-perception and may therefore not accurately reflect actual competence or performance. In addition, knowledge was assessed using items from existing questionnaires, that were reviewed by experts in gerodontology. However, no formal psychometric testing of the questionnaire for nursing staff was performed. Therefore, the reliability and validity of the assessed constructs remain uncertain, e.g. regarding the ability of the instrument to discriminate between different levels of knowledge.

Finally, due to the large amount of missing data from the questionnaires on structural changes in the NHs, we were unable to draw any reliable conclusions on differences between the study groups regarding changes in staffing levels or other organisational factors that could have influenced the implementation of the intervention.

## Conclusion

Our process evaluation provides a comprehensive insight into the implementation of a complex oral health intervention in the NH setting. The interventions’ implementation varied considerably across the nine participating NHs of the IG, with frequent adaptations and inconsistent implementation of remotivation and reinstruction activities. These variations were largely caused by organisational and contextual barriers, such as low staff resources, competing care priorities of the nursing staff, or insufficient managerial or staff support. Additionally, the denial of oral hygiene measures by cognitively impaired or care-resistant residents further limited the reach of the intervention. Overall, the intervention and the regular visits by the DAs were not sufficient to ensure the consistent integration of the dental recommendations into routine practice or to make them widely known among the nursing staff.

Although the intervention concept was generally considered positively by the target groups involved, further approaches may be needed to enhance the intervention, such as educational training for nursing staff and increased hands-on activities by oral health professionals. Furthermore, building a trustful relationship between NH staff and dental professionals may foster greater engagement and support future implementation efforts. The implementation barriers identified in our process evaluation may help to optimise the design and conduct of future complex oral care interventions in the NH setting.

## Supplementary Information

Below is the link to the electronic supplementary material.

Supplementary Material 1

Supplementary Material 2

Supplementary Material 3

Supplementary Material 4

Supplementary Material 5

Supplementary Material 6

## Data Availability

The data used and/or analysed during the current study are available from the corresponding author on reasonable request.

## Abbreviations

NH: Nursing home
cRCT: Cluster-randomised controlled trial
IG: Intervention group
CG: Control group
DA: Dental assistant
SD: Standard deviation
