# Supplementary material for: Process evaluation of a complex intervention to improve the oral health of nursing home residents (MundZaRR): a multimethod study
Source: BMC Geriatr. 2026 Nov 5;26:1406. doi: 10.1186/s12877-026-08607-z (PMC13753250; doi:10.1186/s12877-026-08607-z)
Supplement: Supplementary file 1 [file 12877_2026_8607_MOESM5_ESM.pdf]

Additional file 5. Results of the individual domains of the written surveys

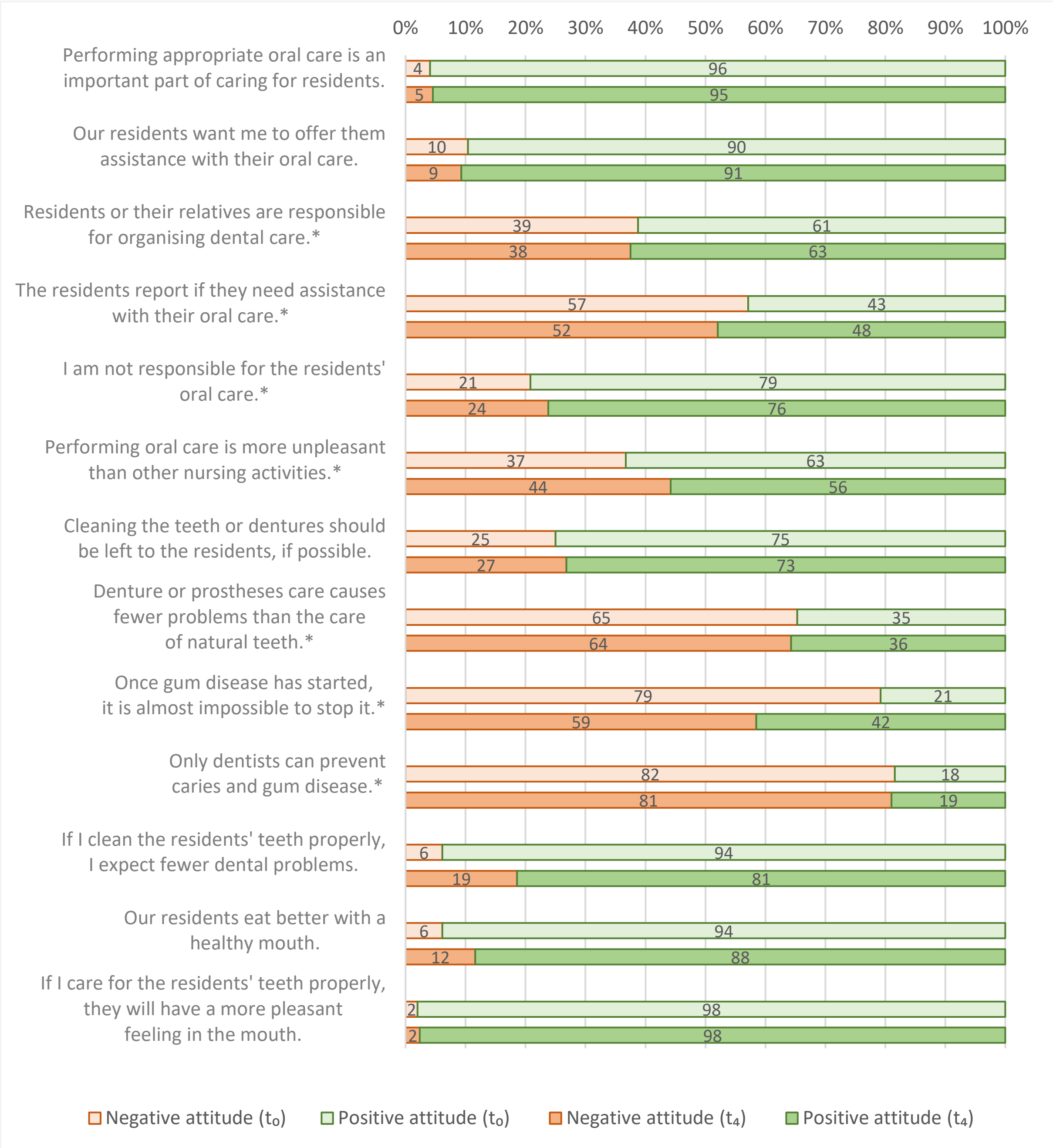

Fig. S2. Attitudes towars oral hygiene in the intervention group at the start and end of the study (For analysis, responses in this domain were dichotomised: ‘strongly agree’ and ‘agree’ were classified as agreement and interpreted as a positive attitude towards oral hygiene, while ‘strongly disagree’ and ‘disagree’ were categorised as disagreement and interpreted as a negative attitude. **Negatively worded items; for these, agreement indicates a negative attitude and disagreement a positive one. These items were inverted for analysis).*

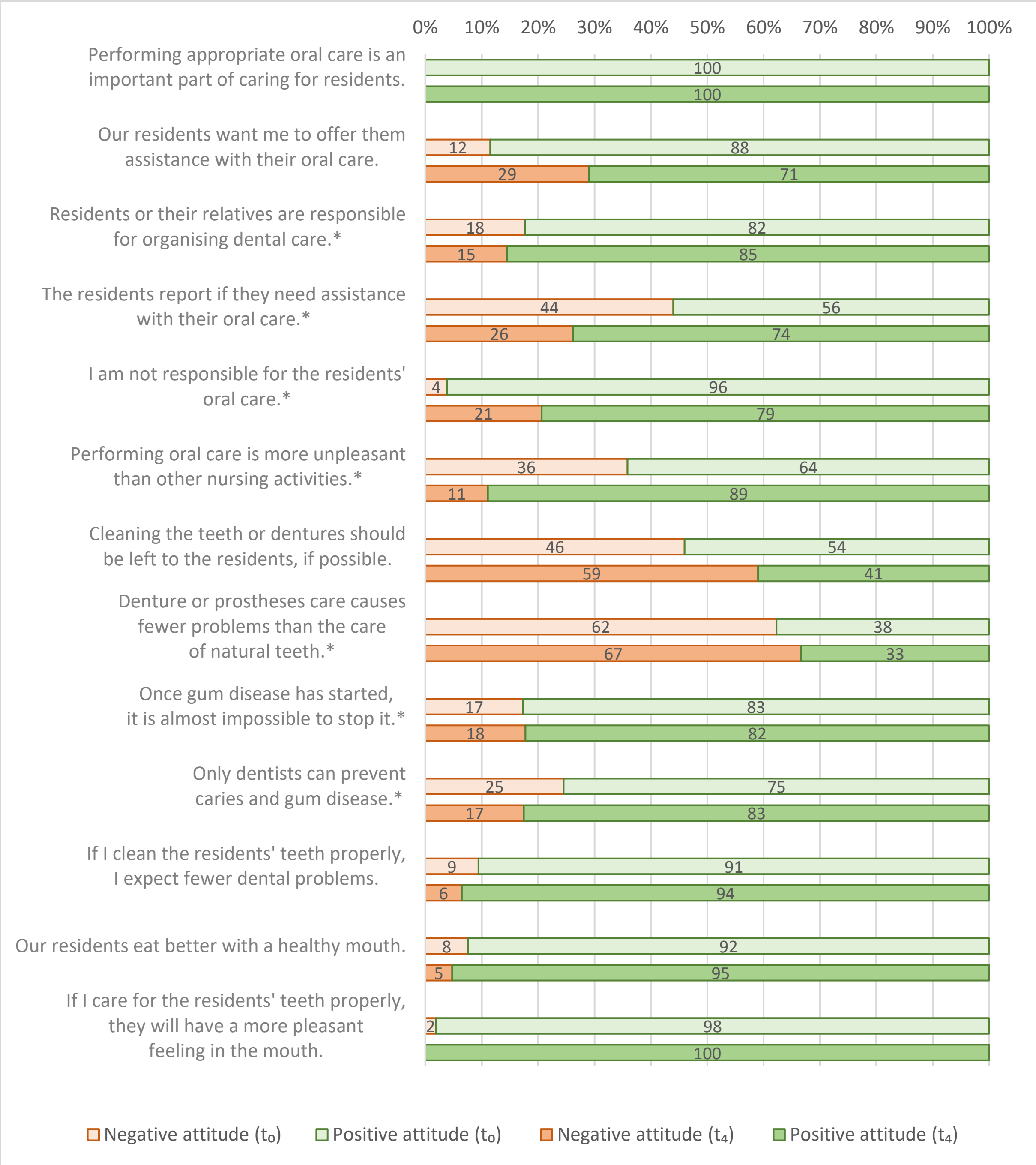

Fig. S3. Attitudes towards oral hygiene in the control group at the start and end of the study (For analysis, responses in this domain were dichotomised: ‘strongly agree’ and ‘agree’ were classified as agreement and interpreted as a positive attitude towards oral hygiene, while ‘strongly disagree’ and ‘disagree’ were categorised as disagreement and interpreted as a negative attitude. **Negatively worded items; for these, agreement indicates a negative attitude and disagreement a positive one. These items were inverted for analysis).*

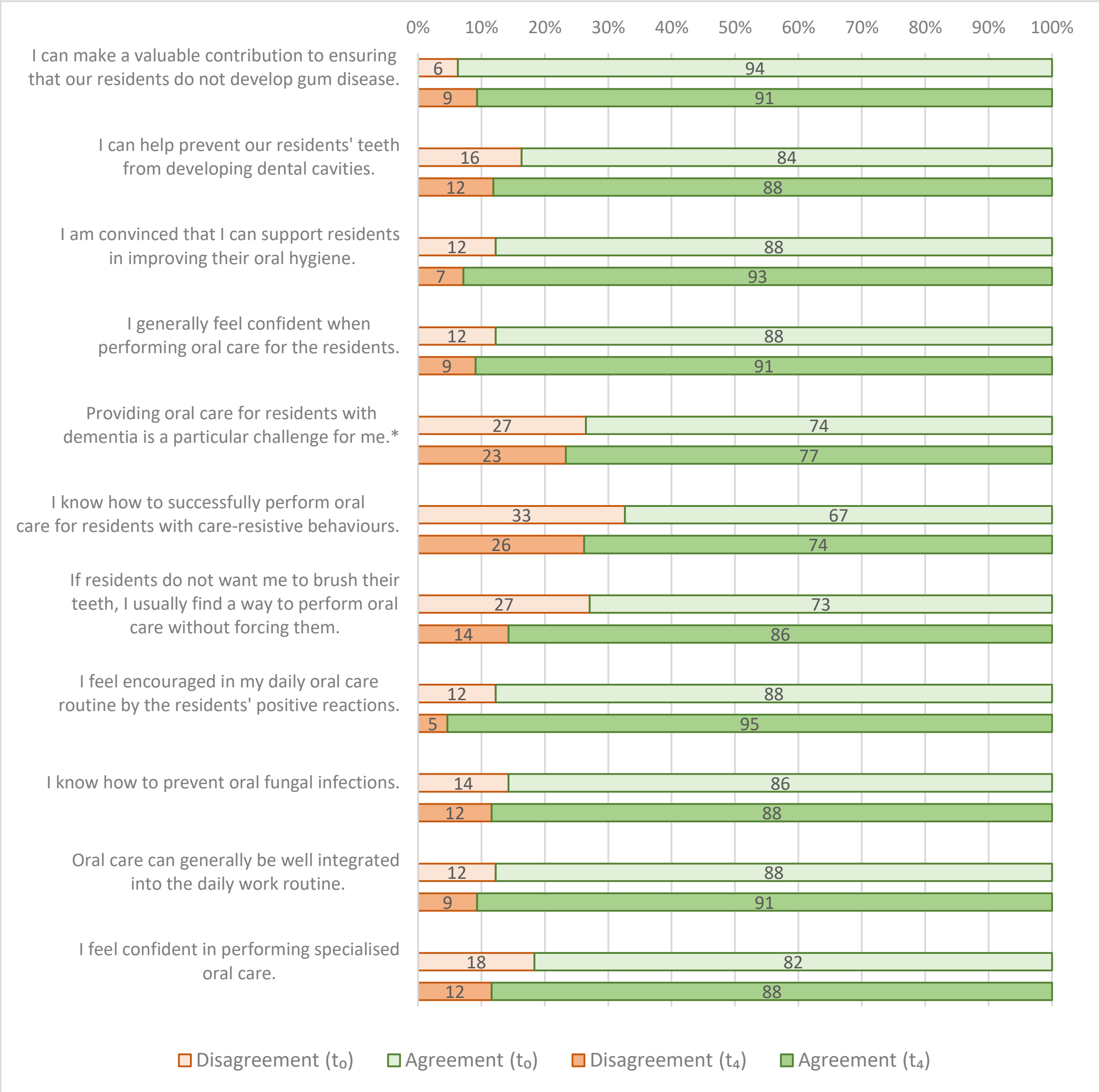

Fig. S4. Self-efficacy regarding oral care in the intervention group at the start and end of the study (For analysis, responses in this domain were dichotomised: ‘strongly agree’ and ‘agree’ were classified as agreement and interpreted as high self-efficacy regarding oral hygiene, while ‘strongly disagree’ and ‘disagree’ were categorised as disagreement and interpreted as low self-efficacy. **Negatively worded item; agreement indicates low self-efficacy and disagreement high self-efficacy).*

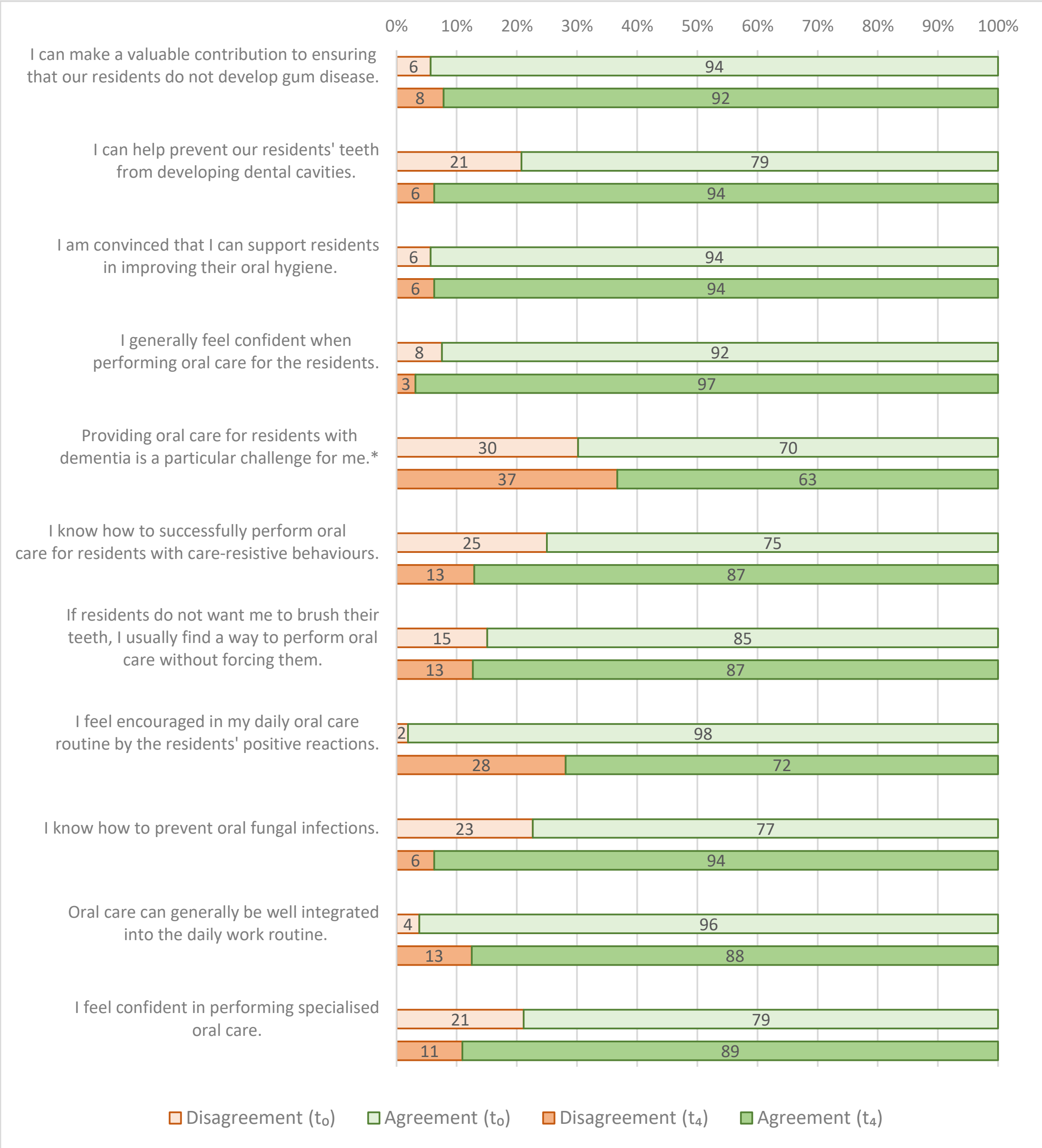

Fig. S5. Self-efficacy regarding oral care in the control group at the start and end of the study. (For analysis, responses in this domain were dichotomised: ‘strongly agree’ and ‘agree’ were classified as agreement and interpreted as high self-efficacy regarding oral hygiene, while ‘strongly disagree’ and ‘disagree’ were categorised as disagreement and interpreted as low self-efficacy. **Negatively worded item; agreement indicates low self-efficacy and disagreement high self-efficacy*).

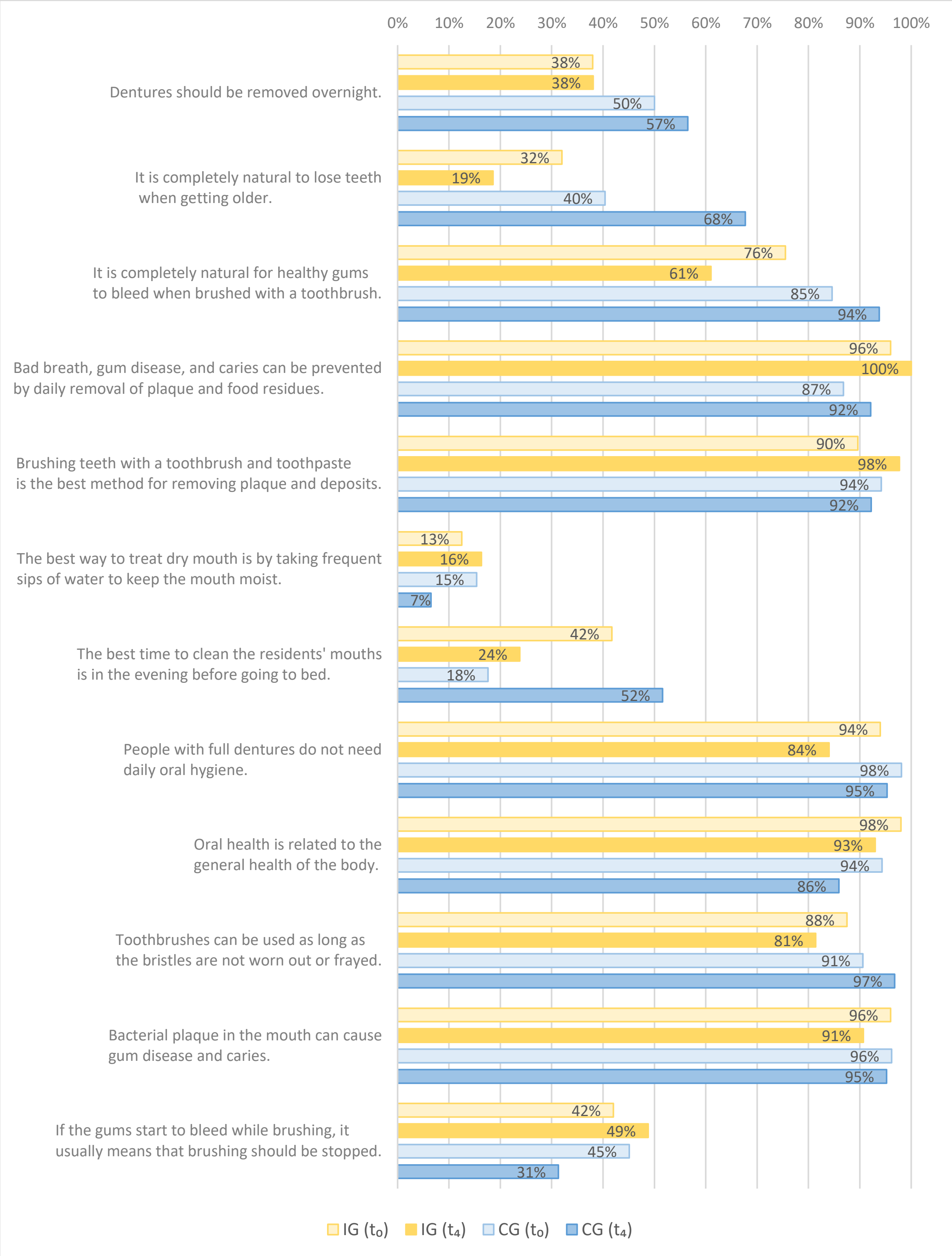

Fig. S6. Knowledge of the intervention and control group regarding oral care at the start and end of the study (frequency of correct answers in %). Abbreviations: IG=intervention group, CG=control group

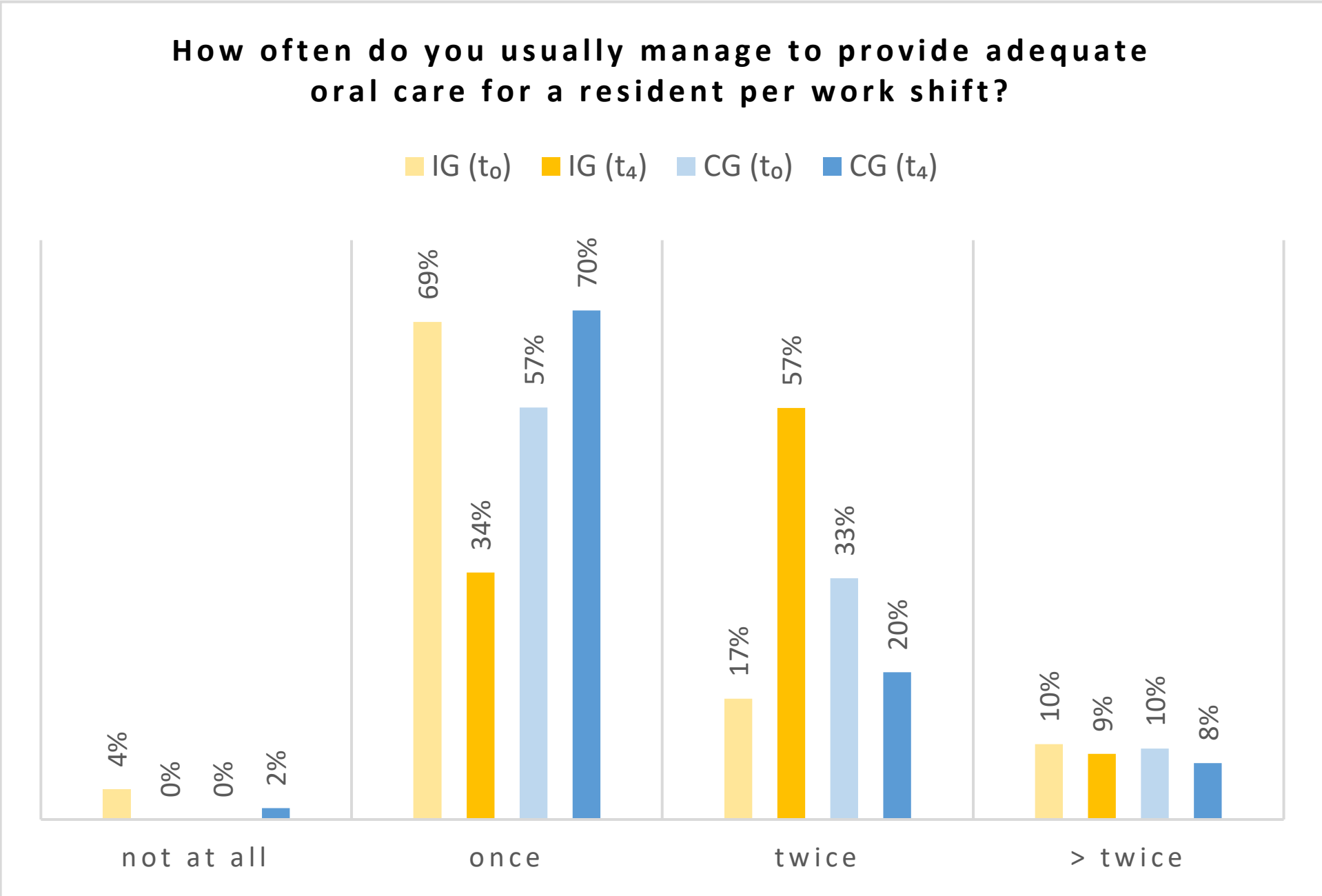

Fig. S7. Frequency of adequate oral hygiene per work shift in the intervention and control group at the start and end of the study. Abbreviations: IG=intervention group, CG=control group

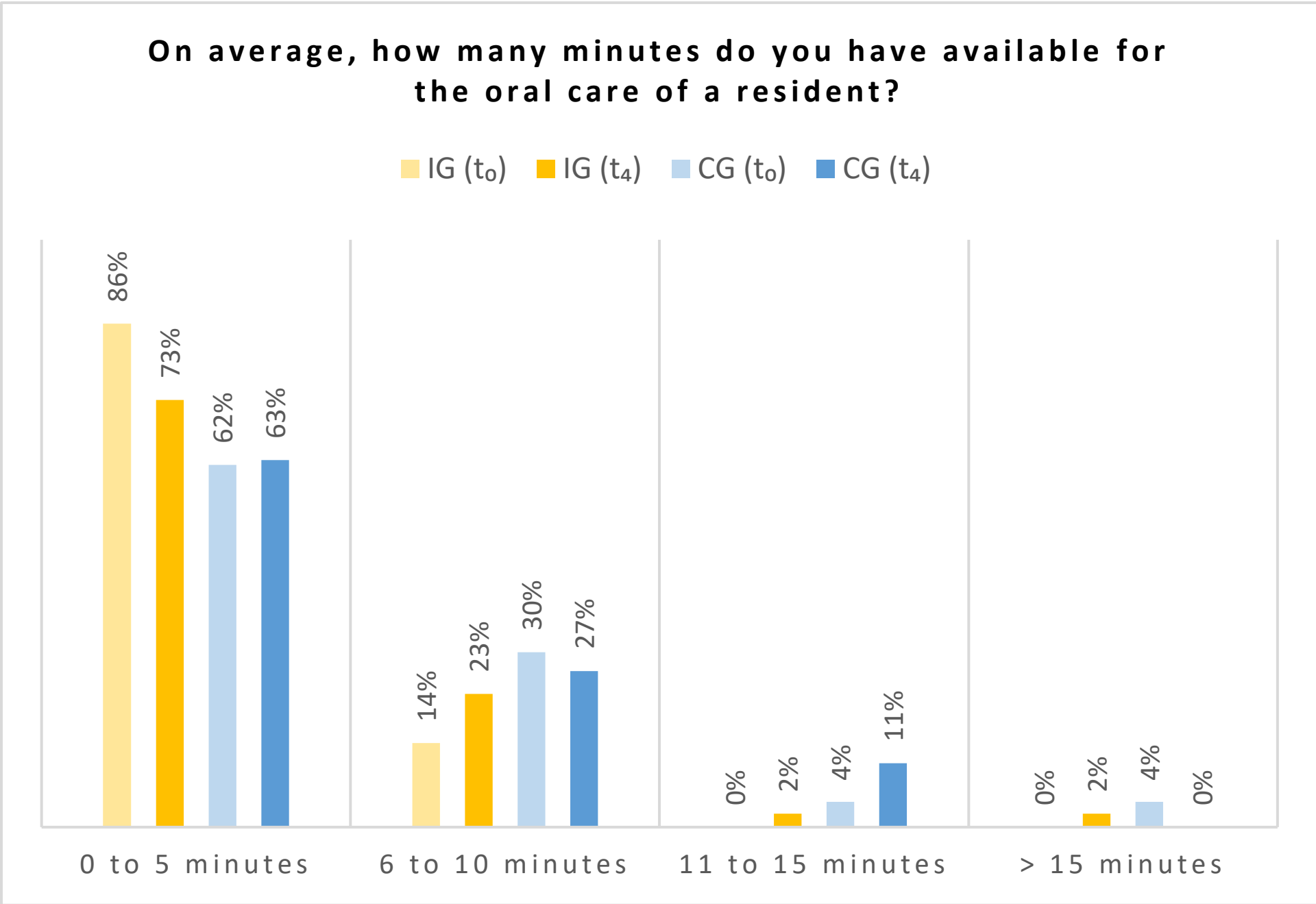

Figure S8. Time available for oral care of residents in the intervention and control group at the start and end of the study. Abbreviations: IG=intervention group, CG=control group

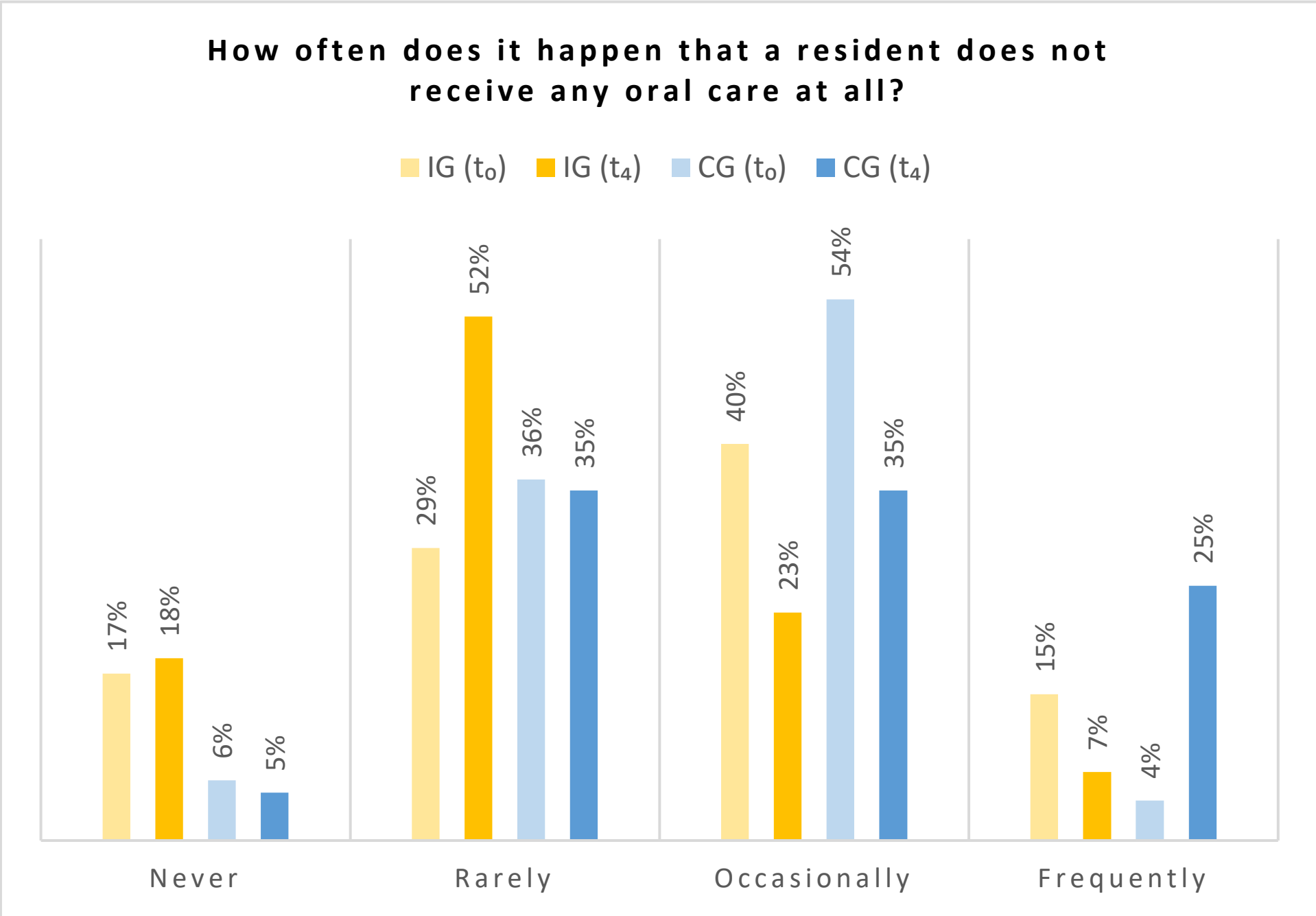

Figure S9. Frequency of not performing any oral care at all in the intervention and control group at the start and end of the study. Abbreviations: IG=intervention group, CG=control group
