# Supplementary material for: Process evaluation of a complex intervention to improve the oral health of nursing home residents (MundZaRR): a multimethod study
Source: BMC Geriatr. 2026 Nov 5;26:1406. doi: 10.1186/s12877-026-08607-z (PMC13753250; doi:10.1186/s12877-026-08607-z)
Supplement: Supplementary file 2 [file 12877_2026_8607_MOESM1_ESM.docx]

**Additional file 1.** Timeline of the MundZaRR study and accompanying evaluations.

**Fig. S1.** Timeline of the MundZaRR cluster-randomised controlled trial, illustrating the intervention procedures and data collection activities of the process evaluation and health economic evaluation. The numbers in the process evaluation timeline correspond to the time points before and after start of the study in months. Abbreviations: cRCT = cluster-randomised controlled trial; DA = dental assistant; NH = nursing home; IG = intervention group; CG = control group.
