# Supplementary material for: Process evaluation of a complex intervention to improve the oral health of nursing home residents (MundZaRR): a multimethod study
Source: BMC Geriatr. 2026 Nov 5;26:1406. doi: 10.1186/s12877-026-08607-z (PMC13753250; doi:10.1186/s12877-026-08607-z)
Supplement: Supplementary file 3 [file 12877_2026_8607_MOESM2_ESM.docx]

Additional file 2. Interview and focus group guides (English translation)

# Interview guide: Study dentists – Intervention group (t_0_)

## Warming-up

- Brief introductions of participants: To start, we would like to ask you to briefly introduce yourself [name, place of residence/catchment area, motivation for participating in the study].

## Opening question

*In the past [x weeks], you have conducted several visits to nursing homes as part of the study to carry out the* ***initial oral examinations*** *of the residents.*

- *Please begin by describing, in general, how these visits have gone so far and what experiences you have had.*

## Initial examinations and interaction with residents

*We would now like to talk with you specifically about the initial examinations of the residents.*

- How did the first examinations of the residents proceed?
  - Where did you conduct the examinations? (Resident's room, corridor, separate room, ...?)
  - Were other people present during the examinations (relatives, caregivers/guardians, roommates, additional nursing staff, ...)? If so, what influence did this have on the examination?
  - How much time did the examinations take per resident on average?
  - On average, how many residents did you examine during one visit to a nursing home?
  - Were the examinations carried out as planned? If not, how did you deal with difficulties or disruptions that arose?
  - How often was it necessary for you to visit the nursing home again because, for example, individual residents were not available?
  - What went particularly well? What did not work well?
  - In your opinion, what could have been done better?
- How did you experience the interaction with the residents so far?
  - Were you confronted with any challenges in this regard? (e.g., challenging behaviour of residents with dementia, difficult access to the oral cavity for bedridden or uncooperative residents) – *If so*, how did you deal with such situations?
- Did the training you received beforehand prepare you well for the examinations?
  - *If not*, what was missing? What information/instructions would you have additionally needed?
- Apart from this study, have you worked in nursing homes in any other professional capacity?
  - *If yes*, what differences did you notice compared with your usual visits?
- Which oral hygiene problems occurred most frequently among the residents?

## Handover of the form / oral hygiene recommendations

*You have just talked about the most common oral hygiene problems among the residents, so now we would like to address your recommendations...*

- In what context did the handover of the form take place?
  - To whom and in what form did you hand over the form? (Was the form simply filed in the documentation folder or discussed with a nursing staff member?)
  - How did you communicate the recommendations to the nursing staff? (Did you discuss the recommendation only with the qualified nursing staff member present at the examination? If not, how many nursing staff did you instruct?)
  - Did you instruct the nursing staff in any other way beyond this? *If so*, what instructions/recommendations did you give?
  - Were there (further) follow-up questions from the nursing staff, or did you have to explain additional aspects?
  - Did you also communicate the results of the examination to the resident and/or their relatives? *If so*, how did that go?
- Which individual oral hygiene recommendations did you give most frequently?
- How realistic do you consider it that the recommendations made will be implemented for the residents?
  - What challenges/barriers do you see regarding implementation?
- In your opinion, what conditions would need to be in place to ensure that your recommendations can be implemented optimally?
- Do you consider the recommendations you provided to be sufficient to improve residents' oral hygiene? *If not,* please briefly explain why not.

## Collaboration and communication with the nursing staff

- How would you describe your collaboration and communication with the nursing staff overall?
- *If not yet addressed*: How did you exchange views with the nursing staff regarding the recommended oral hygiene measures?
- To what extent did you receive support from the nursing staff in dealing with the residents? (helping residents sit up, introducing the dentist and the residents, ...)

## Collaboration with the dental assistants (DAs)

*In one of the training sessions, you got to know the dental assistants (DAs) involved in the study. To what extent have you had contact with the DAs during your recent visits to the nursing home(s)?*

- Were you able to arrange joint appointments for the initial instruction?
  - *If not*, could you explain the reasons for this in more detail?
- How would you describe the collaboration with the DAs?
  - To what extent was the DA involved in the examinations? (Was the DA present at the examination at all? Did she assist, or did she only take an observing role?)
  - To what extent and in what form did you instruct the DAs?
  - Were there any follow-up questions about the form, or did you have to explain things to the DA afterwards?

## Organisation of the on-site visits

*Finally, we would like to discuss with you once again the organisational process of the on-site visits [if applicable: brief recap of the aspects already mentioned].*

- *If nothing has been mentioned so far, start with*: How would you assess the organisation of the visits to the nursing homes so far?
  - How did you perceive the initial contact / scheduling / coordination with the nursing homes?
  - How well could the visits be reconciled with your other professional duties/obligations?
  - To what extent were the residents prepared?
  - To what extent were all parties involved instructed?
  - What (other) organisational barriers/challenges did you experience during the on-site visits? How did you deal with them?
- How did you experience the documentation and handling of the form?
- Were the documentation folders available at the nursing homes/care units?

## Closing

- From your point of view, is there any other feedback or important aspects that we have not addressed, which you would like to add or share with us?

# Interview guide: Study dentists – Control group (t_0_)

## Warming-up

- Brief introductions of participants: To start, we would like to ask you to briefly introduce yourself [name, place of residence/catchment area, motivation for participating in the study].

## Opening question

*In the past [x weeks], as part of the study, you have already conducted several visits for the initial examinations of the residents in the care nursing homes.*

- Please first describe in general terms how these visits have gone so far and what experiences you have had.

## Initial examinations and interaction with residents

- How did the first examinations of the residents proceed?
  - Where did you conduct the examinations? (Resident's room, corridor, separate room, ...?)
  - Were other people present during the examinations (relatives, caregivers/guardians, roommates, additional nursing staff, ...)? *If so*, what influence did this have on the examination?
  - How much time did the examinations take per resident on average?
  - On average, how many residents did you examine during one visit to a nursing home?
  - Were the examinations carried out as planned? If not, how did you deal with difficulties or disruptions that arose?
  - How often was it necessary for you to visit the nursing home again because, for example, individual residents were not available?
  - What went particularly well? What did not work well?
  - In your opinion, what could have been done better?
- How did you experience the interaction with the residents so far?
  - Were you confronted with any challenges in this regard? (e.g., challenging behaviour of residents with dementia, difficult access to the oral cavity for bedridden or uncooperative residents) – *If so*, how did you deal with such situations?
- Did the training you received beforehand prepare you well for the examinations?
  - *If not*, what was missing? What information/instructions would you have additionally needed?
- Apart from this study, have you worked in nursing homes in any other professional capacity?
  - *If yes*, what differences did you notice compared with your usual visits?
- Which oral hygiene problems occurred most frequently among the residents?

## Handover of the form / oral hygiene recommendations

*You have just talked about the most common oral hygiene problems among the residents, so now we would like to address your recommendations...*

- In what context did the handover of the form take place?
  - To whom and in what form did you hand over the form? (Was the form simply filed in the documentation folder or discussed with a nursing staff member?)
  - How did you communicate the recommendations to the nursing staff? (Did you discuss the recommendation only with the qualified nursing staff member present at the examination? If not, how many nursing staff did you instruct?)
  - Did you instruct the nursing staff in any other way beyond this? *If so*, what instructions/recommendations did you give?
  - Were there (further) follow-up questions from the nursing staff, or did you have to explain additional aspects?
  - Did you also communicate the results of the examination to the resident and/or their relatives? *If so*, how did that go?
- Which individual oral hygiene recommendations did you give most frequently?
- How realistic do you consider it that the recommendations made will be implemented for the residents?
  - What challenges/barriers do you see regarding implementation?
- In your opinion, what conditions would need to be in place to ensure that your recommendations can be implemented optimally?
- Do you consider the recommendations you provided to be sufficient to improve residents' oral hygiene? *If not,* please briefly explain why not.

## Collaboration and communication with the nursing staff

*You have already mentioned the communication of the recommendations to the nursing staff; we would now also like to know from you…*

- How did the collaboration and communication with the nursing staff go in general so far?
- *If not yet addressed:* How did you exchange views with the nursing staff regarding the recommended oral hygiene measures?
- To what extent did you receive support from the nursing staff in dealing with the residents? (helping residents sit up, introducing the dentist and the residents, ...)

## Organisation of the on-site visits

*Finally, we would like to discuss with you once again the organisational process of the on-site visits [if applicable: brief recap of the aspects already mentioned].*

- *If nothing has been mentioned so far, start with*: How would you assess the organisation of the visits to the nursing homes so far?
  - How did you perceive the initial contact / scheduling / coordination with the nursing homes?
  - How well could the visits be reconciled with your other professional duties/obligations?
  - To what extent were the residents prepared?
  - To what extent were all parties involved instructed?
  - What (other) organisational barriers/challenges did you experience during the on-site visits? How did you deal with them?
- How did you experience the documentation and handling of the form?
- Were the documentation folders available at the nursing homes/care units?

## Closing

- From your point of view, is there any other feedback or important aspects that we have not addressed, which you would like to add or share with us?

# Focus group guide: Dental assistants (DA) (t_0_)

## Warming-up

- Brief introductions of participants: *To start, we would like to ask you to briefly describe what comes to your mind when you think of the topic “geriatric dentistry.”*

## Initial examinations and collaboration with the dentists

*In one of the training sessions, as well as during the initial examination appointments, you already got to know the study dentists. In this context, we would like to know from you…*

**Opening question: To what extent were you involved in the initial examinations of the residents?**

*If involved,..*

- How would you describe the course of the first examinations of the residents by the dentist?
  - Were you able to arrange joint appointments with the dentists for the first examinations?
    - *If so*, how many appointments could you attend together?
    - *If not,* what were the causes/reasons for this?
  - How would you describe your role during these examinations? (Were you in the same room during the examination? Did you assist, or did you only take an observing role?)
  - How did you perceive the communication with the dentists?
  - Were the dentists' instructions understandable for you and/or the nursing staff?
  - What particularly stands out in your memory from the examinations? (positive/ negative)
- To what extent were you involved in the handover of the form by the dentists?
  - Was the form simply filed in the documentation folder, or discussed with nursing staff members?
  - Did the nursing staff have the opportunity to ask questions? *If so*, which topics/questions were discussed?
  - Did the nursing staff receive any further explanations or instructions from the dentist?
- What other experiences did you have in your collaboration with the dentists? (positive/negative experiences)

## On-site visits and collaboration with the nursing staff

*Following the first examination appointments, you have already conducted on-site visits to the nursing homes as part of the study over the past [x] weeks. We would now like to talk with you specifically about your concrete activities during the on-site visits at the nursing homes…*

**Opening question: How would you describe the course of the first visits to the nursing homes?**

## Reviewing and discussing the form

- From your point of view, how would you describe the joint review of the form with the nursing staff?
  - Did you go through the respective forms together with the nursing staff member on duty/assigned to you? *If so,* where and how did this take place?
  - To what extent did you exchange views with the nursing staff member about this?
  - Were the contents of the forms understandable and comprehensible?
  - Were there any questions or feedback from the nursing staff that you had to clarify, or did you have any questions yourself? *If so,* which ones?
  - Were the forms available for the respective residents at all visits so far? *If not...*
    - How often did it happen that the forms were not available? How did you deal with this situation?
  - Did you get the impression that the nursing staff worked with the forms? To what extent were the contents known to the nursing staff?
  - How did you experience the handling of the form? (Was it a useful working basis for you? Would you have liked additional information/materials?)

## Implementation of the oral hygiene recommendations

- What experiences did you have with the implementation of the oral care measures for the residents?
  - How were the residents involved in the implementation of the measures?
  - How would you describe the interaction between nursing staff and residents?
  - To what extent did the measures carried out correspond to the recommendations from the forms?
  - What were possible reasons why recommendations were not implemented? To what extent did you discuss this with the nursing staff?
- How would you describe the communication between you and the residents?
  - What challenges or problems could you observe in the interaction with the residents, or to what extent were you confronted with them yourself? (e.g., challenging behavior of residents with dementia, difficult access to the oral cavity, for bedridden or uncooperative residents)
- Were you able to provide support to the nursing staff member in this regard? *If so,* how?
- Were there any other problems or challenges affecting the implementation of the measures? (oral care materials not available, disturbances from other people in the room, ...) *If so,* could you explain these in more detail?

## Implementation of remotivation and re-instruction

- What measures/tasks did you carry out as part of **remotivation** in the nursing homes?
  - To what extent were you able to raise the nursing staff's awareness of the topic of oral hygiene in conversations?
  - If you noticed positive developments or a very exemplary oral care situation, how did you communicate this to the nursing staff?
  - To what extent were you able to support problem-solving regarding the implementation of the measures?
  - To what extent were technical questions brought to you, and how were you able to contribute to clarifying these questions?
  - What were factors that helped or hindered you in implementing remotivation well? (structural conditions, disruptive factors, ...)?
- What measures/tasks did you carry out as part of **reinstruction** in the nursing homes?
  - How were you able to support the dissemination of the recommendations from the form to the team?
  - To what extent were you able to support the integration of the oral care measures into the care plan?
  - How were you able to assist the nursing staff in carrying out oral hygiene?
  - What did you notice when reviewing the documentation of completed measures? How did you point out any gaps?
  - How were you able to support the procurement of materials and aids?
  - Which factors were hindering/helpful in carrying out re-instruction?
- Were you able to participate in handovers during your visits? *If so..*
  - Were you able to discuss problems that had occurred? *If so,* what were common problems / what did you discuss, for example?
  - What other topics did you discuss?
- How would you describe the collaboration and communication with the nursing staff in general so far?
  - What feedback/reactions did you receive from the nursing staff regarding your activities (especially in the context of remotivation and re-instruction) during your visits to the nursing homes?
  - How do you assess the nursing staff's acceptance of your recommendations or the measures taken?
  - To what extent were there differing views regarding the implementation of recommended measures?
  - To what extent do you think that the oral hygiene recommendations given will continue to be implemented (or that they will be started after your visit)?
- What differences regarding the aspects just discussed (implementation of remotivation, re-instruction, collaboration with the nursing staff) could you observe between individual care units or between different nursing homes?

## Conclusion of the visits

- Were you able to hold feedback discussions with your main contact persons (e.g. nursing home manager, director of nursing) about the respective course of the day?
  - *If so,* could you explain this in more detail? (How often could these take place? What was discussed? What were the reactions?)

## Organisation of the on-site visits

*Finally, we would like to discuss with you once again the organisational process of the on-site visits [if applicable: brief recap of the aspects already mentioned].*

- *If nothing has been mentioned so far, start with:* How would you describe the organisation of the visits to the nursing homes so far?
  - How did you perceive the initial contact/scheduling /coordination of visits with the nursing homes?
  - To what extent were the residents prepared? To what extent were all parties involved informed about the visit(s)?
  - What influence did the Covid-19 pandemic have on the organisation and implementation of the on-site visits?
- What other organisational barriers/challenges did you experience during the on-site visits? How did you deal with them?
- How did you experience the documentation and handling of the form?
- Were the documentation folders available at the nursing homes/care units?

## Closing

- From your point of view, is there any other feedback or important aspects that we have not addressed, which you would like to add or share with us?

# Interview guide: Nursing home management – Intervention group (t_1_)

## Opening question

*To start, I would like to ask you to briefly describe what comes to mind when you think of oral care or oral health in your residents with care level 3 or above.*

## Importance and practice of oral care/oral health

*The aim of the study is to improve the oral health of residents. Thinking back to the time before the start of the intervention…*

- How would you personally rate the importance of oral care in your nursing home so far?
  - How would you assess the attitude of the nursing staff in your nursing home toward the topic of oral care?
  - What importance do you attach to oral care compared to other care-relevant topics (e.g., pressure ulcer and fall prevention)?
- How was oral care practice performed in your nursing home before participating in the study?
  - Are you familiar with the “Expert Standard for Promoting Oral Health in Nursing Care” published in September 2021?
  - Does the staff in your nursing home work according to this or another standard/concept for oral hygiene? *If so,..*
    - Could you please explain this in more detail?
    - What was done to implement this in the nursing home?
- How would you assess your staff's level of knowledge on the topic of oral care?
  - How would you assess the nursing staff's need for further training/education on the topic of oral hygiene/health?
  - Have the staff in your nursing home already received training on this topic? *If so,* how often and on which topics?
- How does care by external/self-employed dentists take place in the nursing home?
  - Are there regular check-ups?

## Perceived change due to participation in the study

- To what extent have you noticed changes in everyday oral care over the past three months/since the start of the study that you attribute to the study?
  - Who was affected by these changes? What exactly changed? Why did the changes occur?
  - Could you also identify changes that do not relate to oral care?
  - Have you personally noticed any changes in yourself regarding the topic of oral hygiene? (attitude, knowledge, ...)
  - To what extent have you noticed changes over the past three months regarding the nursing staff's level of knowledge about oral care/oral health?
- To what extent have you noticed changes in the oral health of your residents over the past three months? (e.g., improved food intake, fewer inflammations in the mouth, ...)
  - Were there other changes that you noticed?
- What further changes do you hope for in the remaining study period or beyond?
- Do you think that oral hygiene will retain the same importance after the end of the study?

## Implementation of the study

*Next, we are also interested in what experiences you have had over the past months participating in our study.*

- To what extent have you, as [nursing home manager/director of nursing], had contact with the study so far?
  - How much documentation effort was involved? How does the effort compare to the benefit?
- From your point of view, how would you describe the communication and collaboration with the study dentists and dental assistants (DAs) so far?
  - What did you perceive as particularly positive/negative?
  - In which areas did you find the involvement of the DAs (particularly) helpful?
- How do you assess the intervention concept in general?
  - What factors do you perceive as helpful/hindering for the implementation of the intervention?
  - What would you have imagined differently?
  - What suggestions do you have, or what would additionally need to be done to optimise the implementation of the intervention?
- How has the study been perceived so far by the nursing staff and residents?
  - Have you specifically obtained feedback from your staff or residents about the study? *If so,..*
    - In what form? (e.g., conversations with individual staff members, team meetings)
    - What feedback did you receive from the nursing staff or residents?
- What impact has the Covid-19 pandemic had so far on the organisation and implementation of the study in your nursing home? (e.g., regarding obtaining consent, organising visits by the dentists or DAs, visits cancelled due to infections, ...)

## Closing

- What potential do you see in the implementation of the MundZaRR study?
- Is there anything else that seems important to you that has not yet been mentioned?

# Interview guide: Resident council members (t_1_)

## Warming-up

*First of all, we would like to know what first comes to mind when you think of the topic of oral health/oral hygiene in your nursing home.*

## Attitude and stance toward the topic of oral hygiene

*First of all, we would be interested to know…*

- How important is oral care to you personally?
  - What influence does oral care have on your personal well-being?
  - What is/was your attitude toward dental visits in the past? (good/bad relationship with the dentist, anxious patient, ...)
  - Has this attitude changed since you have been living in the nursing home? *If so*, why?

## Practice of oral care and dental treatment in the nursing home

- Do you carry out oral care independently, or is it taken over by the nursing staff?

*If independently:*

- What makes it difficult for you to maintain dental and oral health?
- What would make carrying out oral care easier for you?
- Do nursing staff regularly check with you whether you need support?

*If not independently:*

- In which area of oral care do you receive the most support?
- Do you feel the support provided is sufficient? If not, why not?
- How do you perceive the competencies/skills of the nursing staff regarding oral care?
- How satisfied are you with the oral care practice in this nursing home?
  - How would you assess the importance of the topic of oral hygiene in the nursing home?
  - What is going well? What could be improved?
  - What would you additionally wish for?
- What has your experience been like with accessing dental care?
  - Who do you turn to with dental problems or when you feel you need dental care?
  - Have you had dental problems or conditions of the oral cavity in the past?
  - How did you communicate this to the nursing staff? How was it responded to?
  - The last time you went to the dentist: Were specific recommendations given to you in this regard (e.g., daily use of dental floss, mouthwash, ...)?
    - Did you implement these recommendations, or would you do so? *If not*, what prevents/prevented you from doing so?
  - What problems do you see or are aware of regarding access to dental care? (e.g., problems finding appointments, transport to the dental practice, lack of nursing homes/equipment for an examination on-site, ...)
  - What would make access easier?
- Are there also regular dental check-ups for the residents in the nursing home? If so, how often? By whom? How are they carried out?
- Are you informed about dental/oral care?
  - Do you have sufficient information? If not, what kind of information would you need? How would you like to receive it?
- What services or other offerings would you like to see in order to improve oral health in your nursing home?

## Knowledge on the topic of oral care/oral hygiene

- Why do you think good oral care is important?
- How would you personally rate your level of knowledge on the topic of oral care?
- Would you like more information on the topic of oral care?
  - *If so,* in what form? On which topic specifically?

## Perception of and attitude toward the study

- To what extent have you come into contact with our study so far?

*If known,*

- When and how did you learn about the conduct of the study?
- To what extent have you talked with other residents about the study?
- What do you think about the fact that the topic of oral hygiene is being addressed as part of this study?
- Are you yourself a participant in this study? *If so:*
  - What (positive/negative) changes do you hope for (short- or long-term) (for yourself) as a result of participating in the study?

*If not a study participant:*

- What changes do you hope for in this nursing home as a result of participation in the study, and/or what changes have you already noticed?

*If unknown:*

- What do you think about the fact that the topic of oral hygiene is being addressed as part of this study?
- To what extent have you noticed changes in recent months relating to oral care in this nursing home?

## Closing

- Is there anything else that seems important to you that you would like to share with us?

# Interview guide: Study dentists – Intervention group (t_4_)

## Opening question

*In the interview at the beginning of the study, we already talked about your first visits or data collection appointments at the nursing homes. Now we are interested in how you experienced the subsequent data collection appointments.*

- Please first describe in general terms how these visits went and what experiences you had.

## Examinations and interaction with residents

*We would now like to talk with you in somewhat more detail about the examinations of the residents.*

- To what extent did the follow-up assessments differ from the examinations at the start of the study?
  - Where did you conduct the examinations each time? (Resident's room, corridor, separate room, ...?)
  - Were other people present during the examinations (relatives, caregivers/guardians, roommates, additional nursing staff, ...)? If so, what influence did this have on the examination?
  - How many visits to the nursing homes did you make in total?
  - On average, how much time did the examinations take per resident?
  - On average, how many residents did you examine during one visit to a nursing home?
  - Could the examinations be carried out as planned? *If not,* how did you deal with difficulties or disruptions that arose?
    - How often was it necessary for you to visit the nursing home again because, for example, individual residents could not be reached?
  - What went particularly well? What did not go so well?
  - In your opinion, what could have gone better?
- How did you experience the interaction with the residents?
  - Were you confronted with any challenges in this regard?
  - *If so,* how did you deal with such situations?
  - What, if anything, did you do differently during the follow-up data collection appointments?
- Which oral hygiene problems occurred most frequently among the residents?

## Handover of the form and implementation of the oral hygiene recommendations*.*

- In what context did the handover of the form take place?
  - To whom and how did you hand over the form?
  - How did you communicate the recommendations to the nursing staff?
  - Did you instruct the nursing staff in any other way beyond this? *If so,* what instructions/recommendations did you give?
  - Were there (further) follow-up questions from the nursing staff, or did you have to explain additional aspects?
  - Did you also communicate the results of the examination to the resident and/or their relatives? If so, how did that go?
- Which individual oral hygiene recommendations did you give most frequently?
- In your opinion, how well or how poorly were your recommendations implemented in the individual nursing homes?
  - In your opinion, what were the reasons/factors why recommendations were not implemented? Or what were the factors that promoted good implementation of the recommendations?
  - How realistic do you consider it that the recommendations made will continue to be implemented for the residents even after the end of the study? Could you explain in more detail why or why not?

## Collaboration and communication with the nursing staff

*During your visits to the nursing homes, you certainly also had contact with various nursing staff members. In this context, we would like to know from you…*

- How would you describe your collaboration and communication with the nursing staff in general?
  - Which situations particularly stand out in your memory, positively or negatively?
  - To what extent has the collaboration or communication changed over the course of the study?
- *If not yet addressed:* How did you discussed the recommended oral hygiene measures with the nursing staff?
- To what extent did you receive support from the nursing staff in dealing with the residents? (helping residents sit up, introducing the dentist and the residents, interviewing the residents, ...)

## Collaboration with the dental assistants (DAs)

*Next, I would like to talk with you about the collaboration with the study DA(s).*

- To what extent did you have contact with the DA(s) during your recent visits to the nursing homes?
- How did you perceive the collaboration with the DAs?
  - To what extent was the DA involved in the examinations? (Was the DA present at the examination at all? Did she assist, or did she only take an observing role? To what extent did you receive support from the DA?)
  - To what extent and how did you instruct the DAs?
  - Were there any follow-up questions about the form, or did you have to explain things to the DA afterward?

## Perceived changes over the course of the study

- Over the course of the study, could you identify changes regarding the residents' oral hygiene or oral health?
  - *If so:* Could you describe these in more detail? (e.g., what kind of changes? Who or what was affected?)
  - What might have been possible causes/contributing factors for these changes? (e.g., motivation of the nursing staff, support from nursing home management, …)
- To what extent have you noticed changes regarding oral care practice in the nursing homes?
  - What has changed in the way oral care is carried out? (e.g., use of new tools/materials, new oral care techniques, adapted routines, organisation of training)
  - To what extent do you think these changes will continue even after the end of the project?
- Could you also identify changes that do not relate to oral care? *If so:* Could you please also describe these in more detail?

*If no changes were noticed, or only minor ones:*

- In your view, what were the causes/reasons why no changes occurred?
- What would have (additionally) needed to be done, or what would have been necessary, to achieve changes?

*If not yet specifically addressed:*

- What differences regarding the aspects just discussed (implementation of the oral care recommendations, collaboration with the nursing staff) could you observe between individual care units or between different nursing homes?

## Organisation of the on-site visits

*Finally, we would like to discuss with you once again the organisational process of the on-site visits [if applicable: brief recap of the aspects already mentioned].*

- *If nothing has been mentioned so far, start with:* How do you assess the overall organisation of the visits to the nursing homes?
  - How did you perceive the initial contact/scheduling/coordination of the visits with the nursing homes?
  - How well could the visits be reconciled with your other professional duties/obligations?
  - To what extent were the residents prepared? To what extent were all parties involved informed about the visit?
- What (further) organisational barriers/challenges did you experience during the on-site visits? How did you deal with them?
- How did you experience the documentation and handling of the form?
- What, if anything, would you have wished to be different or additional? (e.g., more support from study staff, better/different preparation for the data collection appointments, …)

## Closing

- From your point of view, is there any other feedback or important aspects that we have not addressed, which you would like to add or share with us?
- What is your final conclusion regarding your participation in the study?

# Interview guide: Study dentists – Control group (t_4_)

## Opening question

*In the interview at the beginning of the study, we already talked about your first visits or data collection appointments at the nursing homes. Now we are interested in how you experienced the subsequent data collection appointments.*

- Please first describe in general terms how these visits went and what experiences you had.

## Examinations and interaction with residents

*We would now like to talk with you in somewhat more detail about the examinations of the residents.*

- To what extent did the follow-up assessments differ from the examinations at the start of the study?
  - Where did you conduct the examinations each time? (Resident's room, corridor, separate room, ...?)
  - Were other people present during the examinations (relatives, caregivers/guardians, roommates, additional nursing staff, ...)? If so, what influence did this have on the examination?
  - How many visits to the nursing homes did you make in total?
  - On average, how much time did the examinations take per resident?
  - On average, how many residents did you examine during one visit to a nursing home?
  - Could the examinations be carried out as planned? *If not,* how did you deal with difficulties or disruptions that arose?
    - How often was it necessary for you to visit the nursing home again because, for example, individual residents could not be reached?
  - What went particularly well? What did not go so well?
  - In your opinion, what could have gone better?
- How did you experience the interaction with the residents?
  - Were you confronted with any challenges in this regard?
  - *If so,* how did you deal with such situations?
  - What, if anything, did you do differently during the follow-up data collection appointments?
- Which oral hygiene problems occurred most frequently among the residents?

## Handover of the form and implementation of the oral hygiene recommendations*.*

- In what context did the handover of the form take place?
  - To whom and how did you hand over the form?
  - How did you communicate the recommendations to the nursing staff?
  - Did you instruct the nursing staff in any other way beyond this? *If so,* what instructions/recommendations did you give?
  - Were there (further) follow-up questions from the nursing staff, or did you have to explain additional aspects?
  - Did you also communicate the results of the examination to the resident and/or their relatives? If so, how did that go?
- Which individual oral hygiene recommendations did you give most frequently?
- In your opinion, how well or how poorly were your recommendations implemented in the individual nursing homes?
  - In your opinion, what were the reasons/factors why recommendations were not implemented? Or what were the factors that promoted good implementation of the recommendations?
  - How realistic do you consider it that the recommendations made will continue to be implemented for the residents even after the end of the study? Could you explain in more detail why or why not?

## Collaboration and communication with the nursing staff

*During your visits to the nursing homes, you certainly also had contact with various nursing staff members. In this context, we would like to know from you…*

- How would you describe your collaboration and communication with the nursing staff in general?
  - Which situations particularly stand out in your memory, positively or negatively?
  - To what extent has the collaboration or communication changed over the course of the study?
- *If not yet addressed:* How did you discussed the recommended oral hygiene measures with the nursing staff?
- To what extent did you receive support from the nursing staff in dealing with the residents? (helping residents sit up, introducing the dentist and the residents, interviewing the residents, ...)

## Perceived changes over the course of the study

- Over the course of the study, could you identify changes regarding the residents' oral hygiene or oral health?
  - *If so:* Could you describe these in more detail? (e.g., what kind of changes? Who or what was affected?)
  - What might have been possible causes/contributing factors for these changes? (e.g., motivation of the nursing staff, support from nursing home management, …)
- To what extent have you noticed changes regarding oral care practice in the nursing homes?
  - What has changed in the way oral care is carried out? (e.g., use of new tools/materials, new oral care techniques, adapted routines, organisation of training)
  - To what extent do you think these changes will continue even after the end of the project?
- Could you also identify changes that do not relate to oral care? *If so:* Could you please also describe these in more detail?

*If no changes were noticed, or only minor ones:*

- In your view, what were the causes/reasons why no changes occurred?
- What would have (additionally) needed to be done, or what would have been necessary, to achieve changes?

*If not yet specifically addressed:*

- What differences regarding the aspects just discussed (implementation of the oral care recommendations, collaboration with the nursing staff) could you observe between individual care units or between different nursing homes?

## Organisation of the on-site visits

*Finally, we would like to discuss with you once again the organisational process of the on-site visits [if applicable: brief recap of the aspects already mentioned].*

- *If nothing has been mentioned so far, start with:* How do you assess the overall organisation of the visits to the nursing homes?
  - How did you perceive the initial contact/scheduling/coordination of the visits with the nursing homes?
  - How well could the visits be reconciled with your other professional duties/obligations?
  - To what extent were the residents prepared? To what extent were all parties involved informed about the visit?
- What (further) organisational barriers/challenges did you experience during the on-site visits? How did you deal with them?
- How did you experience the documentation and handling of the form?
- What, if anything, would you have wished to be different or additional? (e.g., more support from study staff, better/different preparation for the data collection appointments, …)

## Closing

- From your point of view, is there any other feedback or important aspects that we have not addressed, which you would like to add or share with us?
- What is your final conclusion regarding your participation in the study?

# Focus group guide: Dental assistants (t_4_)

## Examinations and collaboration with the dentists

*In the first group interview at the start of the study, we already discussed your appointments for data collection/examination of the residents together with the study dentists. In this context, we would like to know from you…*

**Opening question: To what extent were you involved in the subsequent examination appointments (in February/March) of the residents?**

- To what extent has the process of the examinations changed compared to the examinations at the start of the study?
  - Did you also take part in the second examination appointments with the dentists? If not, what were the causes/reasons for this?
  - *If so,* what tasks did you have during the examinations? (Were you in the same room during the examination? Did you assist, or did you only take an observing role?)
- To what extent were you involved in the handover of the form by the dentists?

*If involved,…*

- How did you experience these handovers? (Were you involved in the handover? Was the form simply filed in the documentation folder, or discussed with a nursing staff member? What exactly was discussed?)
- Did the nursing staff have the opportunity to ask questions? If so, which topics/questions were discussed?
- Did the nursing staff receive any further explanations or instructions from the dentist?
- Did you yourself have any questions for the dentist that you asked regarding the form during the handover? If so, which topics/questions were discussed?
- How did you experience the collaboration and communication with the dentists?
  - What particularly stands out in your memory regarding the collaboration, positively or negatively?
  - To what extent were you in contact with the dentists?

## On-site visits and collaboration with the nursing staff

*Over the course of the past year, you have conducted various on-site visits to the care nursing homes as part of the study. We would now like to talk with you once again about your concrete activities during the on-site visits at the nursing homes…*

**Opening question: How would you describe a typical course of the (follow-up) visits to the nursing homes?**

## Reviewing and discussing the form

- To what extent did you discuss the forms together with the nursing staff?
  - Did you go through the respective forms together with the nursing staff member on duty/assigned to you? If so, where and how did this take place? (Access to the forms, in what environment and with whom was the discussion held, …)
  - Were the forms available for the respective residents at all visits? *If not…*
    - How often did it happen that the forms were not available? How did you deal with this situation?
  - Did you get the impression that the nursing staff worked with the forms? To what extent were the contents and recommendations known to the nursing staff?
  - How did you experience the handling of the form and the documentation folders?
    - Would you have liked additional information/materials? If so, which ones?
    - Were the documentation folders available at the nursing homes/care units? To what extent were they fully or incompletely maintained?

### Implementation of remotivation and re-instruction

- What measures/tasks did you carry out as part of **remotivation** in the nursing homes?
  - To what extent were you able to raise the nursing staff's awareness of the topic of oral hygiene in conversations?
  - If you noticed positive developments or a very exemplary oral care situation, how did you communicate this to the nursing staff?
  - To what extent were you able to support problem-solving regarding the implementation of the measures?
  - To what extent were technical questions brought to you, and how were you able to contribute to clarifying these questions?
  - What were factors that helped or hindered you in implementing remotivation well? (structural conditions, disruptive factors, ...)?
- What measures/tasks did you carry out as part of **reinstruction** in the nursing homes?
  - How were you able to support the dissemination of the recommendations from the form to the team?
  - To what extent were you able to support the integration of the oral care measures into the care plan?
  - How were you able to assist the nursing staff in carrying out oral hygiene?
  - What did you notice when reviewing the documentation of completed measures? How did you point out any gaps?
  - How were you able to support the procurement of materials and aids?
  - Which factors were hindering/helpful in carrying out re-instruction?
- Were you able to participate in handovers during your visits? *If so..*
  - Were you able to discuss problems that had occurred? *If so,* what were common problems / what did you discuss, for example?
  - What other topics did you discuss?
- How would you describe the collaboration and communication with the nursing staff in general so far?
  - What feedback/reactions did you receive from the nursing staff regarding your activities (especially in the context of remotivation and re-instruction) during your visits to the nursing homes?
  - How do you assess the nursing staff's acceptance of your recommendations or the measures taken?
  - To what extent were there differing views regarding the implementation of recommended measures?
  - To what extent do you think that the oral hygiene recommendations given will continue to be implemented (or that they will be started after your visit)?
- What differences regarding the aspects just discussed (implementation of remotivation, re-instruction, collaboration with the nursing staff) could you observe between individual care units or between different nursing homes?

### Collaboration and communication with the nursing staff

- How did you perceive the collaboration and communication with the nursing staff in general?
  - What feedback/reactions did you receive from the nursing staff regarding your activities (especially in the context of remotivation and re-instruction) during your visits to the nursing homes?
  - How do you rate the nursing staff's acceptance of your recommendations or the measures taken?
  - To what extent were there differing views regarding the implementation of recommended measures?
  - Which situations particularly stand out in your memory, positively or negatively?
  - Were you able to hold feedback discussions with your main contact persons (head of nursing/nursing home management) about the respective course of the day? *If so,* could you explain this in more detail? (How often could these take place? What was discussed? What were the reactions of the head of nursing/nursing home management?)

### Implementation of the oral hygiene and treatment recommendations

- What experiences did you have with the implementation of the oral care and treatment recommendations for the residents?
  - How were the residents involved in the implementation of the measures? (e.g., discussion of treatment needs, recommended materials, etc.)
  - How did the interaction between the nursing staff/you and the residents take place when implementing the recommendations?
    - What challenges or problems could you observe when dealing with the residents, or to what extent were you confronted with them yourself? (e.g., challenging behavior, for instance in residents with dementia, difficult access to the oral cavity, for instance in bedridden or uncooperative residents) How did you deal with this?
  - To what extent did the measures carried out correspond to the recommendations from the forms?
  - What were possible reasons why recommendations or treatments were not carried out, or were deviated from?
    - Were there any other problems or challenges affecting the implementation of the measures? (e.g., aids not available, disturbances from other people in the room, rejection of the treatment/recommendations by the resident/relatives/treating dentist) If so, could you explain these in more detail?
- To what extent do you think that the oral hygiene recommendations given will continue to be implemented in the future?

### Perceived changes over the course of the study

- Over the course of the study, what changes could you identify regarding the residents' oral hygiene or oral health?
- What might have been possible causes/contributing factors for the changes? (e.g., motivation of the nursing staff, support from nursing home management, …)
- To what extent have you noticed changes regarding oral care practice in the nursing homes?
- What has changed in the way oral care is carried out? (e.g., use of new tools/materials, new oral care techniques, adapted routines, organisation of training)
- To what extent do you think these changes will continue even after the end of the project?
- What further changes could you identify that do not relate to oral care?
- Could you describe these in more detail? (e.g., what kind of changes? Who or what was affected?)

*If no changes were noticed, or only minor ones:*

- In your view, what were the causes/reasons why no changes occurred?
- What would have (additionally) needed to be done, or what would have been necessary, to achieve changes?

*If not yet specifically addressed:*

- What differences regarding the aspects just discussed (implementation of remotivation, re-instruction, and the oral care recommendations, collaboration with the nursing staff) could you observe between individual care units or between different nursing homes?

## Organisation of the on-site visits

*Finally, we would like to discuss with you once again the organisational process of the on-site visits [if applicable: brief recap of the aspects already mentioned]…*

- *If nothing has been mentioned so far, start with:* How do you rate the organisation of the visits to the nursing homes?
  - How did you perceive the initial contact/scheduling /coordination of your visits with the nursing homes?
  - To what extent were the residents and other parties involved (nursing home contact persons, nursing staff) present, prepared, or informed during the visits?
  - What influence did the Covid-19 pandemic have on the organisation and implementation of the on-site visits? (e.g., visiting bans, residents not available, nursing homes difficult to reach)
- What other organisational barriers/challenges did you experience during the on-site visits? How did you deal with them?

## Closing

- From your point of view, is there any other feedback or important aspects that we have not addressed, which you would like to add or share with us?
- What is your final conclusion regarding your participation in the study?
