# Supplementary material for: Process evaluation of a complex intervention to improve the oral health of nursing home residents (MundZaRR): a multimethod study
Source: BMC Geriatr. 2026 Nov 5;26:1406. doi: 10.1186/s12877-026-08607-z (PMC13753250; doi:10.1186/s12877-026-08607-z)
Supplement: Supplementary file 4 [file 12877_2026_8607_MOESM3_ESM.docx]

**Additional file 3.** Sample characteristics of the participatory observations

| **Characteristics** |  |
| --- | --- |
| **Nursing homes (n=3)** | |
| Ownership, *n* |  |
| Church-run | 2 |
| Public | 1 |
| Location, *n* |  |
| rural | 1 |
| urban | 2 |
| Size, *n* |  |
| 50-100 beds | 2 |
| > 100 beds | 1 |
| **Residents (n=20)** | |
| Gender, *n (%)* |  |
| Female | 17 (85) |
| Male | 3 (15) |
| Age, *mean [min;max], years* | 81.5 [64;94] |
| Care level, *n (%)* |  |
| 3 | 10 (50) |
| 4 | 6 (30) |
| 5 | 4 (20) |
| Diagnoses/limitations *(multiple answers possible*), *n (%)* |  |
| Dementia | 12 (60) |
| Immobility | 4 (20) |
| Stroke | 3 (15) |
| Need for assistance with oral hygiene^1^, *n (%)* |  |
| None | 4 (20) |
| Partial | 7 (35) |
| Full | 9 (45) |
| **Nursing staff (n=8)** | |
| Gender, *n (%)* |  |
| Female | 7 (88) |
| Male | 1 (12) |
| Age, *mean [min;max], years* | 44.5 [22;67] |
| Professional qualification, *n* |  |
| Registered nurse with university degree | 2 |
| Registered nurse without university degree |  |
| General nurse | 2 |
| Geriatric nurse | 2 |
| Nursing assistant |  |
| General nursing assistant | 1 |
| Geriatric nursing assistant | 1 |
| Work experience in geriatric care, *mean [min;max], years* | 18.3 [1;37] |
| Years working in the current nursing home, *mean [min; max], years* | 10.3 [1;26] |
| ^1^ as rated by a study dentist at main data collection |  |
