# Supplementary material for: Process evaluation of a complex intervention to improve the oral health of nursing home residents (MundZaRR): a multimethod study
Source: BMC Geriatr. 2026 Nov 5;26:1406. doi: 10.1186/s12877-026-08607-z (PMC13753250; doi:10.1186/s12877-026-08607-z)
Supplement: Supplementary file 5 [file 12877_2026_8607_MOESM4_ESM.docx]

**Additional file 4.** Sample characteristics of registered nurses from the written surveys at study start and end

|  | **t_0_** | | **t_4_** | |
| --- | --- | --- | --- | --- |
| **Characteristics** | **Intervention group  (n=50)** | **Control group**  **(n = 53)** | **Intervention group**  **(n=44)** | **Control group**  **(n=64)** |
| Gender*, *n (%)* |  |  |  |  |
| Female | 35 (71) | 36 (77) | 30 (70) | 51 (82) |
| Male | 14 (29) | 10 (21) | 11 (26) | 10 (16) |
| Non-binary | – | 1 (2) | 2 (4) | 1 (2) |
| Age group*, *n (%)* |  |  |  |  |
| < 20 years | 2 (4) | 1 (2) | 1 (2) | 1 (2) |
| 20 to 29 years | 9 (19) | 11 (22) | 12 (27) | 15 (24) |
| 30 to 39 years | 20 (42) | 16 (33) | 14 (32) | 19 (30) |
| 40 to 49 years | 6 (12) | 8 (16) | 12 (27) | 11 (17) |
| > 50 years | 11 (23) | 13 (27) | 5 (12) | 17 (27) |
| Employment status*, *n (%)* |  |  |  |  |
| Full-time | 30 (65) | 31 (63) | 18 (42) | 40 (64) |
| Part-time | 16 (35) | 18 (37) | 25 (58) | 23 (36) |
| Average weekly working hours, *[min;max]* | 32 [28;35] | 24 [16;30] | 28 [16;37] | 28 [16;35] |
| Professional role in the nursing home, *n (%)* |  |  |  |  |
| Geriatric nurse | 27 (54) | 26 (49) | 18 (41) | 35 (55) |
| General nurse | 5 (10) | 8 (15) | 5 (11) | 10 (16) |
| Geriatric nursing assistant | 16 (32) | 16 (30) | 18 (41) | 18 (28) |
| General nursing assistant | 2 (4) | 3 (6) | 3 (7) | 1 (1) |
| Work experience in geriatric care*, *mean [min;max], years* | 12.9 [1;32] | 13.6 [1;40] | 10.3 [1;38] | 15.3 [2;40] |
| Participation in training on oral care*, *n (%)* |  |  |  |  |
| Yes | 11 (25) | 4 (9) | 10 (23) | 6 (10) |
| No | 33 (75) | 42 (91) | 33 (77) | 52 (90) |
| * not answered by all participants; **Measurement points**: t_0_ = study start; t_4_= after 12 months | | | | |
