# Supplementary material for: Process evaluation of a complex intervention to improve the oral health of nursing home residents (MundZaRR): a multimethod study
Source: BMC Geriatr. 2026 Nov 5;26:1406. doi: 10.1186/s12877-026-08607-z (PMC13753250; doi:10.1186/s12877-026-08607-z)
Supplement: Supplementary file 6 [file 12877_2026_8607_MOESM6_ESM.docx]

**Additional file 6.** Sample characteristics of the individual and focus group interviews

| **Characteristics** |  |
| --- | --- |
| **Dentists (n=7)** | |
| Gender, *n (%)* |  |
| Male | 6 (86) |
| Female | 1 (14) |
| Age*, mean [min; max], years* | 56.7 [40;67] |
| Specialist dentist training *(multiple answers possible)*, *n* |  |
| Oral surgery | 1 |
| Periodontology | 1 |
| Endodontology | 1 |
| Environmental dentistry | 1 |
| Conservative dentistry | 1 |
| Implantology | 1 |
| Not applicable | 3 |
| Current employment status, *n (%)* |  |
| Self-employed |  |
| In solo practice | 3 (43) |
| In a group practice | 1 (14) |
| In a medical care centre | 1 (14) |
| Employed |  |
| In a joint practice | 1 (14) |
| Locum | 1 (14) |
| **Dental assistants (n=3)** | |
| Gender, *n (%)* |  |
| Female | 3 (100) |
| Male | 0 (0) |
| Age*, mean [min; max], years* | 28.3 [26;31] |
| Professional Qualification, *n* (%) |  |
| Dental assistant | 3 (100) |
| Additional qualifications, *n* |  |
| Prophylaxis assistant | 1 |
| Dental billing | 1 |
| Work experience, *mean [min; max], years* | 5.0 [3;7] |
| Employment besides study participation, *n* |  |
| Dental assistant in a solo practice | 1 |
| Not applicable | 2 |

**Additional file 6**. Sample characteristics and the individual and focus group interviews (continued)

| **Characteristics** | |
| --- | --- |
| **Nursing home managers (n=7)** | |
| Gender, *n* *(%)* |  |
| Female | 7 (100) |
| Male | 0 (0) |
| Age, *mean [min; max], years* | 40.9 [33;57] |
| Professional qualification, *n* *(%)* |  |
| Registered nurse with university degree | 1 (14) |
| Registered nurse without university degree |  |
| General nurse | 2 (29) |
| Geriatric nurse | 4 (57) |
| Additional qualifications *(multiple answers possible),* *n* |  |
| Nursing home manager | 1 |
| Head of nursing service | 3 |
| Ward manager | 2 |
| Practice instructor | 1 |
| De-escalation trainer | 1 |
| Quality management | 1 |
| Current leadership role, *n (%)* |  |
| Nursing home manager | 1 (14) |
| Nursing service manager | 4 (57) |
| Deputy nursing service manager | 1 (14) |
| Ward/area manager in geriatric care | 1 (14) |
| Work experience in leadership roles, *mean [min; max], years* | 8.0 [2;15] |
| **Nursing staff (n=5)** | |
| Gender, *n* (%) |  |
| Female | 4 (80) |
| Male | 1 (20) |
| Age, *mean [min; max], years* | 34.6 [28;42] |
| Professional qualification, *n* (%) |  |
| Registered nurse without university degree |  |
| Geriatric nurse | 4 (80) |
| Nursing assistant |  |
| Geriatric nurse assistant | 1 (20) |
| Work experience in geriatric care, *mean [min; max], years* | 12.0 [8;17] |
| Years working in the nursing home, *mean [min; max], years* | 9.0 [1;14] |
